# Phenotypic divergence is driven by mobile genetic elements in a heritable insect symbiont

**DOI:** 10.1073/pnas.2607226123

**Published:** 2026-09-21

**Authors:** Balig Panossian, Matthew R. Kolp, Taoping Wu, Keertana Tallapragada, Vilas Patel, Elliott B. Goldstein, Kerry M. Oliver, Lee M. Henry, Benjamin J. Parker

**Affiliations:** ^a^ School of Biological and Behavioural Sciences, Queen Mary University of London, London E1 4NS, United Kingdom; ^b^ Department of Microbiology, University of Tennessee, Knoxville, TN 37996; ^c^ Department of Biology, University of North Carolina at Chapel Hill, Chapel Hill, NC 27599; ^d^Department of Entomology, University of Georgia, Athens, GA 30602

**Keywords:** symbiosis, mobile genetic elements, pathogenic fungi, horizontal gene transfer

## Abstract

Heritable microbes profoundly influence insect biology, yet the traits they confer often evolve rapidly and differ among closely related symbiont strains. Despite their importance, we lack a clear understanding of how novel traits arise in symbionts and how this diversity influences host ecology in nature. The aphid facultative symbiont *Regiella insecticola* is ideally suited to address this question due to strong lineage-specific variation in host benefits. By generating 20 high-quality genomes, we found that *Regiella*’s evolution is driven largely by gene gains mediated by mobile genetic elements (MGEs). A plasmid (pRILSR1) encoding a type IV secretion system and a highly expressed predicted effector has spread horizontally between distantly related *Regiella* clades associated with pea aphids. Notably, only pRILSR1-bearing strains confer protection against the fungal pathogen *Pandora neoaphidis*. Moreover, loss of the plasmid by a protective strain in culture resulted in the loss of protection, indicating that pRILSR1 is required for the defensive phenotype. In a multiyear field study, pRILSR1 frequency varied systematically among host plant-associated pea aphid populations and predicted differences in symbiont-mediated fungal resistance. Together, our results show that gain and loss of a single MGE contributes to divergence in a key adaptive trait, providing a mechanism by which symbiont evolution generates phenotypic differences among host populations.

## Significance

Heritable symbionts can rapidly reshape host ecology, yet the mechanisms generating phenotypic variation among natural populations remain poorly understood. Although some symbiont-conferred traits are encoded on plasmids and phages, whether these elements spread adaptive host-benefiting traits across symbiont lineages on eco-evolutionary timescales remains untested. We show that a horizontally exchanged plasmid is essential for a symbiont to protect its host against a fungal pathogen and that variation in plasmid carriage tracks differences in resistance among sympatric host populations. Together, our results indicate that mobile elements structure ecologically relevant differences in symbiont-conferred traits, providing a mechanism for rapid, population-level divergence.

Symbioses between microbes and eukaryotes are ubiquitous and central to host biology (1). Yet, the evolutionary processes that generate and maintain functional diversity in these associations remain poorly understood. At one extreme, open microbial systems such as animal gut communities experience frequent gene exchange, with mobile genetic elements (MGEs) driving rapid phenotypic change (2). At the other, obligate vertically transmitted symbionts evolve under limited gene flow and strong genetic drift, leading to genome reduction and functional decay (3).

Many symbioses fall between these extremes, where vertical transmission promotes gene loss, yet occasional horizontal transfer introduces new genes that fuel functional innovation (4–6). This pattern is exemplified by heritable facultative symbionts of insects, which are not essential to host survival and often occur at intermediate frequencies in natural populations (7). Key factors shaping the persistence and spread of heritable symbionts are the ecologically relevant phenotypes they confer on insect hosts, including benefits such as protection against natural enemies and costly traits such as reproductive manipulation (5). Critically, these phenotypes vary even among closely related symbiont strains, suggesting that symbionts frequently gain and lose relevant genes (8–12). These phenotypes are often encoded on MGEs, including plasmids and bacteriophages (13, 14). For example, cytoplasmic incompatibility in *Wolbachia* and *Rickettsia*, male-killing and defensive toxins in *Spiroplasma*, and parasitoid protection in *Hamiltonella defensa* are all associated with MGEs (15–21). These examples highlight the potential for MGEs to shape ecologically important host traits, yet their role in generating phenotypic variation among natural populations remains poorly understood.

Aphids and their symbionts provide a powerful system to address this question. In addition to their obligate symbiont *Buchnera aphidicola*, which supplies essential nutrients missing from plant phloem (22), aphids harbor several facultative symbionts that are primarily vertically transmitted but can also move horizontally between host lineages (23). *Regiella insecticola* (hereafter *Regiella*) stands out for its diverse, lineage-specific ecological benefits, including defense against fungal pathogens (24, 25), parasitoid wasp protection (26), resistance to a viral pathogen (27), and improved performance on certain host plants (28). In pea aphids (*Acyrthosiphon pisum*), *Regiella* is consistently associated with specific sympatric plant-adapted aphid populations (called “biotypes”) (29–31). Yet, how *Regiella* acquires and diversifies the genes underlying these benefits, and whether this variation contributes to ecological differentiation among host populations, remains unknown.

In this study, we combined genome sequencing of cultured *Regiella* strains with laboratory assays and a multiyear field study to investigate whether MGEs spread adaptive traits between symbiont lineages, and how this shapes protection in natural host populations. We found that MGEs have played a major role in *Regiella’s* evolution, including the horizontal spread of a small plasmid (pRILSR1) among distantly related symbiont clades associated with pea aphids. In laboratory assays, pRILSR1 was uniformly associated with protection against the fungal pathogen *Pandora neoaphidis* (hereafter, *Pandora*). Consistent with this finding, a strain that lost the plasmid in culture lost protection, implicating pRILSR1 in the defensive phenotype. In the field, pRILSR1 frequency differed among plant-adapted aphid populations and predicted their fungal resistance. Together, our study provides the most in-depth analysis to date of how the dynamics of MGEs govern symbiont evolution and confer adaptive traits across host populations.

## Results

### *Regiella* Is Highly Genetically Diverse as Shown by Multilocus Sequence Type (MLST) Analysis.

We first used an MLST scheme to characterize the genetic diversity of *Regiella* associated with diverse aphid species [based on (32)]. We detected 346 *Regiella*-positive aphids from 23 different aphid species (Dataset S1), from which we identified 119 unique sequence types. Qualitatively, we found that some *Regiella* strains are restricted to a single aphid species, while others span multiple species (*SI Appendix*, Fig. S1). Similarly, some aphid species harbor only a single strain of *Regiella* while others carry multiple strains (*SI Appendix*, Fig. S2). These patterns suggest that *Regiella* symbionts are gained and lost among aphid lineages at relatively high frequencies.

### Culturing and Parallel Sequencing Approaches Produce Highly Complete *Regiella* Genomes.

Based on the MLST screen, we selected phylogenetically diverse *Regiella* strains for genome sequencing that either belonged to a supported *Regiella* clade (*SI Appendix*, Fig. S1) or had existing phenotypic data (33–35). We used a culturing technique followed by long- or short-read sequencing to produce high-quality genomes for 20 *Regiella* strains (Table 1). Four additional strains were sequenced but removed from the study due to insufficient genome coverage. This approach yielded highly complete chromosomal assemblies and a composite reference database of known phage and plasmid sequences for identifying MGEs. Most assemblies exhibited high CheckM completeness scores (>92.2%). Two strains from *Drepanosiphum aceris* aphids (strains 2027 and 2033) had substantially smaller genomes (1.36 Mbp) and lower GC content (39.47%), suggesting a distinct evolutionary history (*SI Appendix*, *Supplementary Results*). Among the assemblies, we identified 18 plasmids clustered into four types along with 20 complete bacteriophages (Dataset S2), most of which were shared among multiple *Regiella* strains (see below). For genome analyses, we also included the previously sequenced TUt *Regiella* strain (35).

**Table 1.** Genome statistics and metadata for *Regiella* strains analyzed in this study

Strain	Aphid host species	Host plant	Genome size (Mbp)	GC%	Genome Complete-ness% (CheckM)	Sequencing technology	In vitro cultured	Clade	MLST type
LSR	*A. pisum*	*Medicago sativa*	2.2	42.46	98.88	PacBio	+	1	1
MD10	*A. pisum*	*Medicago* sp.	1.97	42.04	98.88	Illumina	+	1	1
MeliA18	*A. pisum*	*Melilotus alba*	2.57	42.48	96.69	PacBio	++	2	115
CF7	*A. pisum*	*Trifolium* sp.	1.95	42.06	96.54	Illumina	N/A	2	116
313	*A. pisum*	*Trifolium* sp.	2.55	42.46	96.69	PacBio	++	2	116
T8	*A. pisum*	*Trifolium* sp.	2.58	42.51	96.69	PacBio	++	2	116
T5	*A. pisum*	*Trifolium* sp.	2.57	42.47	96.69	PacBio	++	2	116
RU4	*Aphis fabae*	*Rumex* sp.	2.26	42.47	97.77	Illumina	N/A	4	111
2027	*D. aceris*	*Acer campestre*	1.36	39.46	84.65	Illumina	N/A	5	119
2033	*D. aceris*	*Acer campestre*	1.36	39.47	84.88	Illumina	N/A	5	119
Y1	*Macrosiphoniella millefolii*	*Achillea millefolium*	2.66	42.61	96.50	Illumina	N/A	3	118
R3	*Macrosiphum rosae*	*Rosa* sp.	2.63	42.57	98.74	Illumina	N/A	3	118
GAH	*Macrosiphum sp.*	*Galium* sp.	1.44	41.78	92.21	Illumina	N/A	1	112
GE1	*Macrosiphum sp.*	*Geum* sp.	2.03	42.27	98.88	Illumina	N/A	1	113
GE2	*Macrosiphum sp.*	*Geum* sp.	2.02	42.26	98.88	Illumina	N/A	1	113
327	*Metopolophium sp.*	*Phragmites australis*	1.91	42.09	94.01	Illumina	N/A	2	82
440	*Metopolophium sp.*	*Phragmites australis*	1.91	42.09	94.01	Illumina	N/A	2	82
5.15	*Myzus persicae*	*Hirschfeldia incana*	2.53	42.95	98.80	PacBio	++	4	111
RR5	*Sitobion avenae*	*Glyceria maxima*	2.53	42.23	97.10	Illumina	−	4	111
Gr2	*Sitobion sp.*	*Glyceria maxima*	1.89	42.06	95.01	Illumina	N/A	3	117

**Fig. 1.**
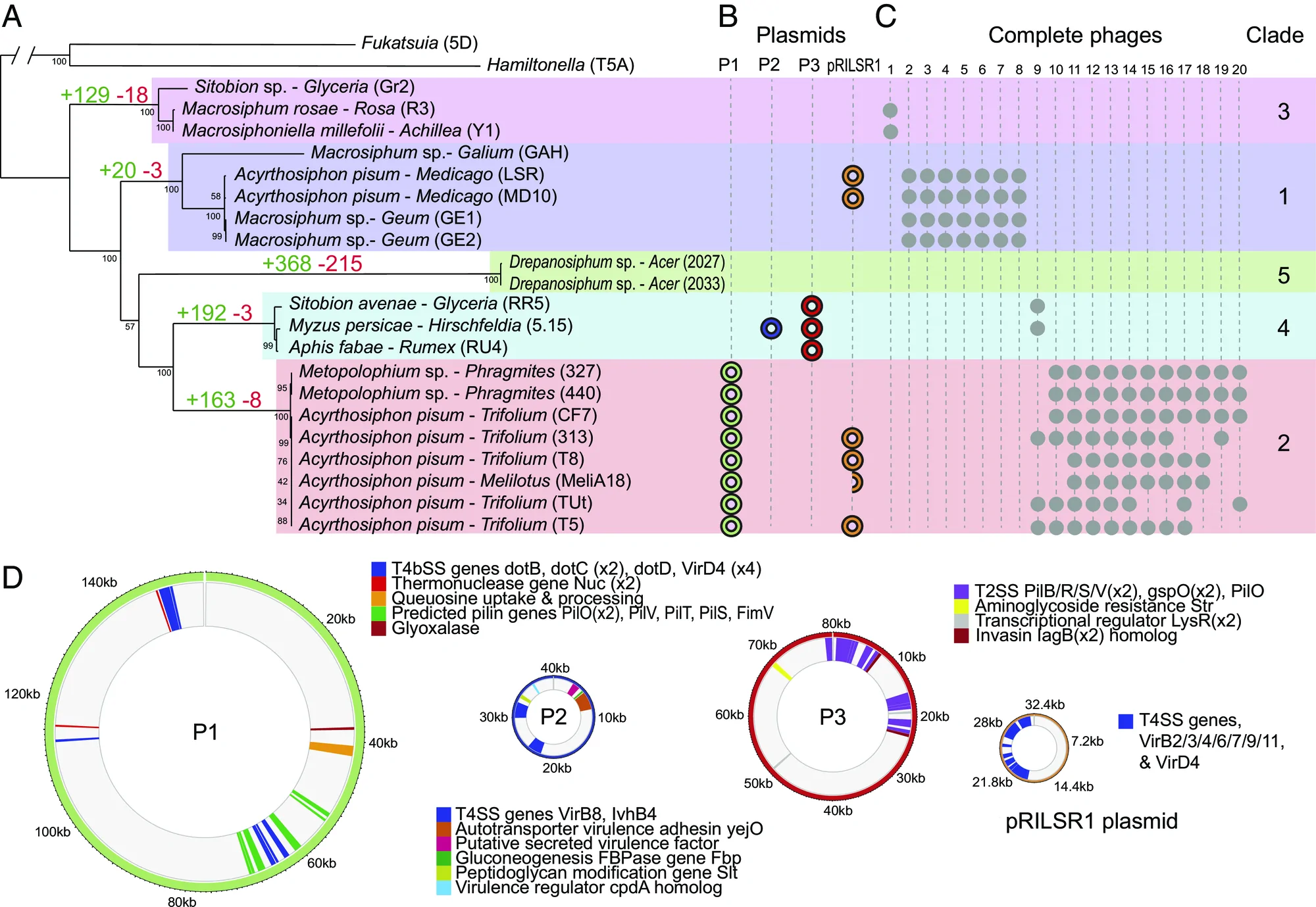
*Regiella* forms distinct phylogenetic clades with characteristic complements of MGEs. (*A*) Maximum-likelihood phylogenetic tree of all *Regiella* genome sequences based on amino acid sequences of 147 universal single copy orthologous genes. The tree is rooted to *Fukatsuia symbiotica* strain 5D (GCA_003122425.1) and *H. defensa* strain T5A (GCA_000021705.1). Tip names represent the aphid species, the host plant genus, and the aphid clone unique identifier that each *Regiella* strain was collected from. Strains of *Regiella* are clustered into five distinct clades, each labeled with the number of genes uniquely present (green) or absent (red) in all strains of a particular clade. Each clade is shaded with a different color and clade numbers are indicated on the right side of the figure. (*B*) Plasmid presence across *Regiella* genomes is shown, with identities labeled at the *Top*. (*C*) Complete bacteriophages identified in each *Regiella* genome. Phages were grouped according to shared coding sequences. (*D*) Shows the four annotated plasmids identified in the *Regiella* genomes, with gene functional categories based on annotation shown in colors as indicated in the keys.

For each sequenced *Regiella* genome, we indicate strain name, aphid host species, host plant from which the aphid was collected, basic genome assembly metrics including genome size, GC%, and completeness, the sequencing technology employed, in vitro culturing details (++ indicates adequate bacterial growth, + indicates minimal growth, − indicates no growth, N/A indicates not attempted), phylogenetic clade (matching Fig. 1), and MLST type (Dataset S1).

### *Regiella* has Diversified into Multiple Genetically Distinct Clades and has Moved Horizontally Across Aphid Species.

We identified five well-supported *Regiella* clades using a phylogeny based on 147 single-copy orthologous gene sequences from the genome assemblies (Fig. 1*A*). Consistent with previous studies (32, 36), *Regiella* strains from different pea aphid biotypes fell into two distinct clades, and strains from these clades were also detected in other aphid species. *Regiella* from clade 1 included strains from the *M. sativa* and other pea aphid biotypes (*SI Appendix*, Fig. S1 and Dataset S1) grouped with strains from *Macrosiphum* species. *Regiella* strains from clade 2, which are typically found in *Trifolium* and *Melilotus* pea aphid-biotypes (32), were also identified in *Metopolophium* aphids. These relationships, together with high amino acid sequence similarity between *Regiella* from pea aphids and non-pea aphid hosts, indicate horizontal transfer of *Regiella* strains among distantly related aphid species. The remaining clades are described in *SI Appendix*, *Supplementary Results*.

### *Regiella* Genome Evolution Is Driven by Gene Gains Across Clades.

A pangenome analysis revealed a conserved core genome shared across all sequenced strains alongside distinct accessory gene sets that have been differentially gained or lost among clades (Fig. 1*A* and *SI Appendix*, Fig. S3 and Dataset S3). Core genome content was largely consistent across the *Regiella* phylogeny, except in strains isolated from *D. aceris*, which have undergone genome erosion. We found many genes uniquely present within each clade, but very few clade-specific losses (Fig. 1*A* and *SI Appendix*, Fig. S4), indicating that gene gain has had a greater impact than gene loss on *Regiella* diversification. Annotation of clade-specific genes using COG, KEGG, GO, and Pfam databases (Dataset S4 and *SI Appendix*, Fig. S4 and *Supplementary Results*) revealed that each *Regiella* clade carries characteristic complements of bacteriophages (Fig. 1*B*), plasmids (Fig. 1*C*), and transposable elements (*SI Appendix*, Fig. S5), pointing to MGEs as primary drivers of gene gains across the phylogeny.

### Plasmids are a Major Source of Gene Gains Across the *Regiella* Phylogeny.

Our culture-based approach enabled identification of four plasmid types shared among strains (Fig. 1*D*), most of which were missing from previous *Regiella* genome assemblies. The largest, P1 (~145 kb), encodes multiple predicted pilin genes, and was restricted to *Regiella* clade 2 from *Trifolium* and *Melilotus* biotype pea aphids and *Metopolophium* aphids; it was present in the TUt assembly (35), but previously reported as part of a chromosomal contig. A second plasmid, P2 (~40 kb), was unique to the previously sequenced strain 5.15 (clade 4) from *Myzus* aphids and encodes Type IV secretion system genes, a putative secreted virulence factor, and a cpdA homolog. A third ~80 kb plasmid (P3), absent from the original 5.15 genome assembly (33), was present in all three clade 4 strains and encodes for a Type II secretion system and two copies of IagB, a homolog of a gene implicated in eukaryotic cell invasion. Full plasmid gene annotations are provided in *SI Appendix*, *Supplementary Results* and *Datasets* (functional assignments are based on sequence similarity and conserved domains).

### The pRILSR1 Plasmid Is Shared by Distantly Related *Regiella* Clades.

The smallest plasmid (~32 kb, called pRILSR1), first reported in *Regiella* strain LSR (34), was exclusively associated with *Regiella* strains from pea aphids (Fig. 1*A*). pRILSR1 is present in *Regiella* strains from clades 1 and 2 despite their phylogenetic distance, suggesting horizontal transfer of the plasmid between *Regiella* strains. This plasmid encodes a Type IV secretion system (T4SS) including VirD4 and VirB2 through VirB11. We found that pRILSR1 is absent from some pea aphid strains (CF7 and TUt) as well as from all non-pea aphid-associated *Regiella* in clades 1 and 2, including strains from *Macrosiphum* and *Metopolophium* aphids. One pea aphid strain, MeliA18, retained only a partial copy of the plasmid that contained 20 out of 48 genes. With the exception of MeliA18, the pRILSR1 sequences found in clade 1 and 2 strains of *Regiella* were highly similar, ranging from 96 to 99% AAI (amino acid identity). Together, these patterns indicate that pRILSR1 has spread horizontally among pea aphid-associated *Regiella* but is gained and lost at the strain level.

### The pRILSR1 Plasmid Is Implicated in Fungal Protection.

**Fig. 2.**
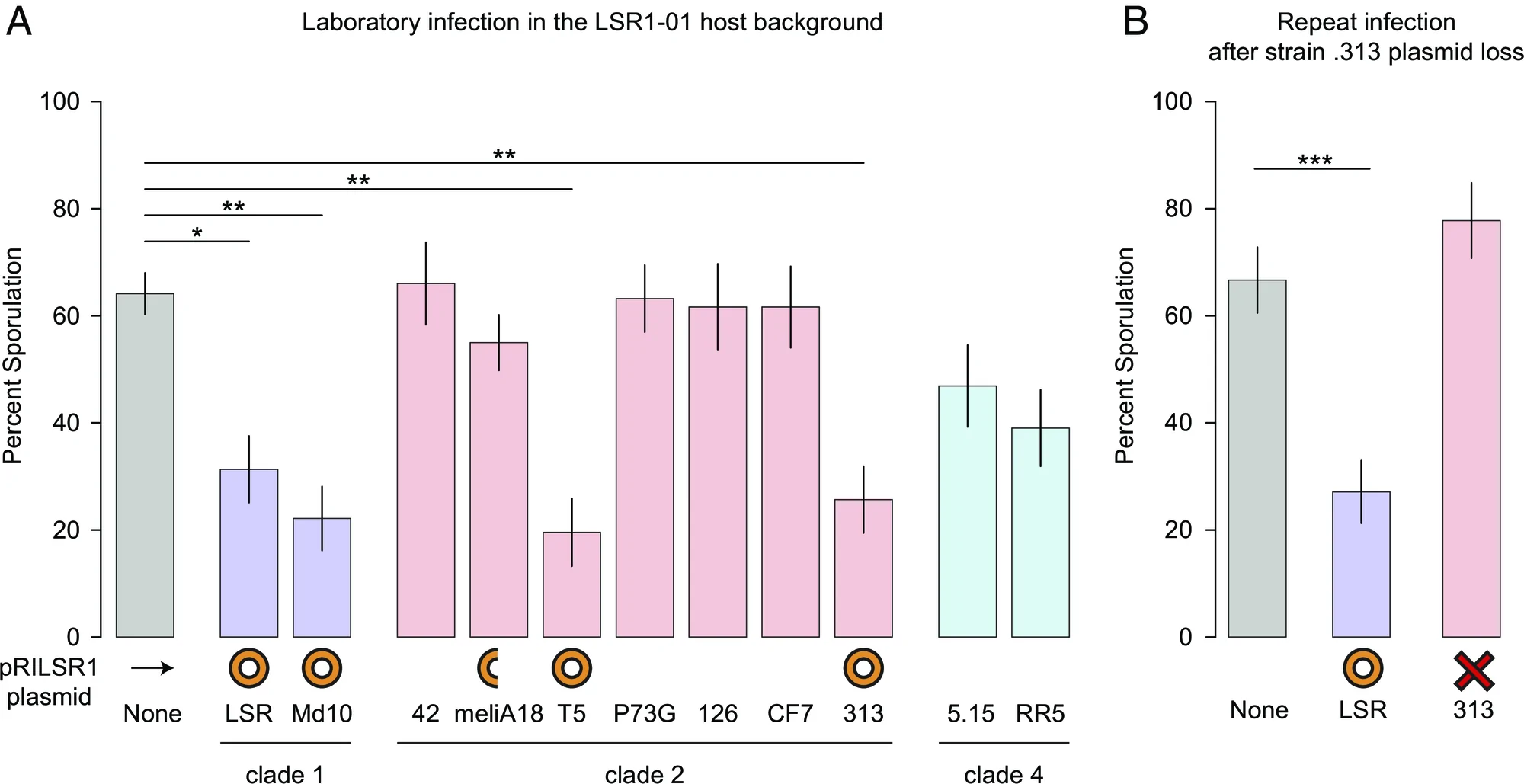
Laboratory *Pandora* infection of *Regiella* strains in a common host genetic background implicates pRILSR1 in fungal protection. (*A*) The y-axis shows the percent of aphids that developed a characteristic fungal cadaver, indicating infection with *Pandora*. Symbiont strains and clades are shown along the x-axis, with pRILSR1 presence indicated with an orange circle. Control aphids without *Regiella* are shown in gray to the left of the figure. Error bars show SE, and statistical significance is shown along the top of the figure (* < 0.05; ** < 0.01; *** < 0.001). (*B*) Shows a repeat of this infection after strain 313 lost the pRILSR1 plasmid. The y-axis again shows percent sporulation, and error bars show SE.

We established 11 *Regiella* strains from three clades into a common aphid genetic background [LSR1-01; (37)] and challenged each line with the fungal pathogen *P. neoaphidis*. Four strains conferred significant protection against fungal infection (Fig. 2*A* and *SI Appendix*, *Supplementary Results*), measured as a reduction in the proportion of exposed aphids producing sporulating cadavers. All four protective strains carried pRILSR1, whereas every strain lacking the plasmid, as determined by genome sequencing or diagnostic PCR screening, conferred no protection (Fig. 2*A*).

To test whether pRILSR1 is required for protection, we took advantage of a natural plasmid loss event in strain 313 during laboratory culture (*SI Appendix*, *Supplementary Results*). We used Nanopore sequencing to confirm strain identity and the complete loss of pRILSR1: Across 677 single-copy BUSCO genes, the polished assembly was 99.998% identical to the reference at the nucleotide level, and no reads mapped to the plasmid. We challenged strain 313 with *Pandora* following plasmid loss and found that it had lost protection (Fig. 2*B*), demonstrating that pRILSR1 is required for the protective phenotype. Two additional lines of evidence reinforce this conclusion: Protective and nonprotective clade 2 *Regiella* have nearly identical chromosomal genomes (99.97 to 99.99% AAI) but differ in pRILSR1 presence, and strain MeliA18, which carries only a partial copy of the plasmid is not protective. Together, these results indicate that pRILSR1 underlies *Regiella*-mediated fungal protection.

### pRILSR1 Predicts Fungal Resistance in Field-Collected Aphids.

**Fig. 3.**
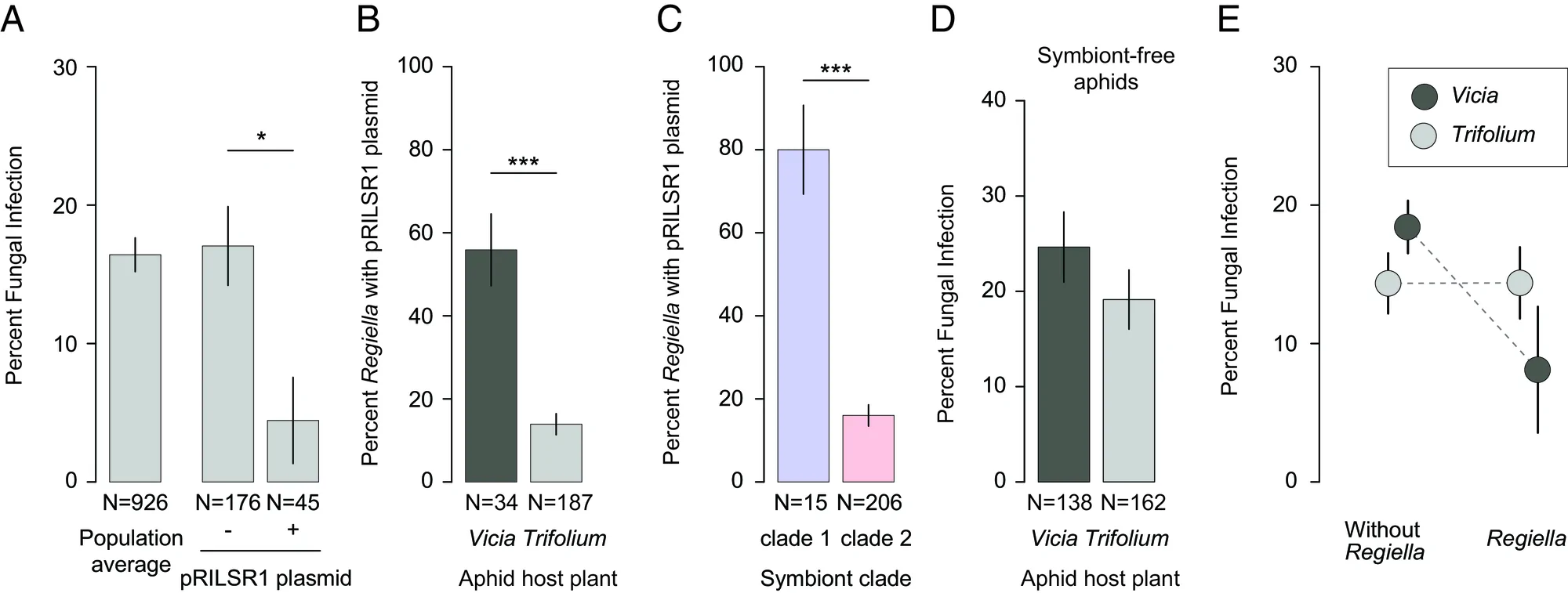
Field data link the pRILSR1 plasmid to variation in *Pandora* protection. (*A*) The y-axis shows the percent of aphids that developed a fungal cadaver when brought into the lab. Samples are grouped into the entire population (population average), aphids with *Regiella* that do not have the pRILSR1 plasmid (−), and aphids with *Regiella* that harbor the pRILSR1 plasmid (+); sample sizes are shown along the bottom of the figure. (*B*) shows the percent of *Regiella* from aphids collected from *Vicia* and *Trifolium* that harbored the pRILSR1 plasmid. (*C*) shows the same information for *Regiella* from clades 1 vs. 2. (*D*) Shows the percent of aphids that developed a fungal infection, showing only symbiont-free (no facultative symbionts of any species) from *Vicia* vs. *Trifolium*. (*E*) Shows the strength of *Regiella*-mediated protection in aphids collected from *Vicia* vs. *Trifolium* host plants. The y-axis was calculated as [1 – (sporulation with symbiont/sporulation without symbiont)] *100, with complete protection against fungus conferred by a symbiont at the top of the figure and no protection at the *Bottom*. In each panel, error bars show SE, and statistical significance is shown along the top of the figure (* < 0.05; ** < 0.01; *** < 0.001).

A 2-y field study revealed that carriage of the pRILSR1 plasmid, rather than *Regiella* presence alone, predicts fungal protection in natural populations. We collected nearly 1,000 aphids from multiple host plants, recorded whether each produced a *Pandora* cadaver after 5 d in the lab (Dataset S5), and screened each individual for facultative symbionts and pRILSR1. Overall, 16.4% of aphids developed a *Pandora* infection, yet harboring *Regiella* had no significant effect on fungal infection (χ^2^ = 0.62, 923 df, *P* = 0.43); a result likely explained by only 20.4% of *Regiella* strains carrying the plasmid. Aphids with pRILSR1-positive *Regiella* were significantly less likely to develop a fungal infection than those carrying pRILSR1-negative *Regiella* (Fig. 3*A*; χ^2^ = 5.71, 1 df, *P* = 0.017). The plasmid had no effect on wasp parasitism (χ^2^ = 0.62, 1 df, *P* = 0.43) or mortality from unidentified causes (χ^2^ = 0.022, 1 df, *P* = 0.88).

### pRILSR1 Frequency Tracks Divergence in Pathogen Protection Across Sympatric Host Populations.

Pea aphids in our field study were collected from multiple host plants, including species of *Trifolium* (n = 471) and *Vicia* (n = 455). Overall, the frequency of pRILSR1 differed significantly among sympatric pea aphid populations. The plasmid was more common in *Regiella* from *Vicia*-feeding aphids than from *Trifolium*-feeding aphids (Fig. 3*B*; χ^2^ =16.0, 1 df, *P* < 0.0001), and more common in clade 1 than clade 2 *Regiella* (Fig. 3*C*; χ^2^ = 27.1, 1 df, *P* < 0.0001). *Pandora* prevalence did not differ among symbiont-free aphids across biotypes (Fig. 3*D*; χ^2^ = 1.32, 1 df, *P* = 0.25), indicating similar pathogen pressures. However, the protective effect of *Regiella* on fungal infection varied between biotypes (Fig. 3*E*; host plant x *Regiella* interaction; χ^2^ = 3.4, 920 df, *P* = 0.065), although this was only marginally significant. To test whether plasmid carriage contributes to biotype-associated variation in fungal protection, we compared a model including host plant and *Regiella* presence/absence with a model in which *Regiella* infections were partitioned by pRILSR1 status. Incorporating plasmid status significantly improved model fit (χ^2^ = 6.21, 1 df, *P* = 0.013), demonstrating that plasmid carriage explains fungal protection beyond host plant and *Regiella* presence alone. Together, these results demonstrate that variation in pRILSR1 frequency contributes to divergence in symbiont-mediated fungal protection between *Vicia*- and *Trifolium*-adapted pea aphid populations.

### A Predicted T4SS Effector Is Highly Expressed from the Protective pRILSR1 Plasmid.

**Fig. 4.**
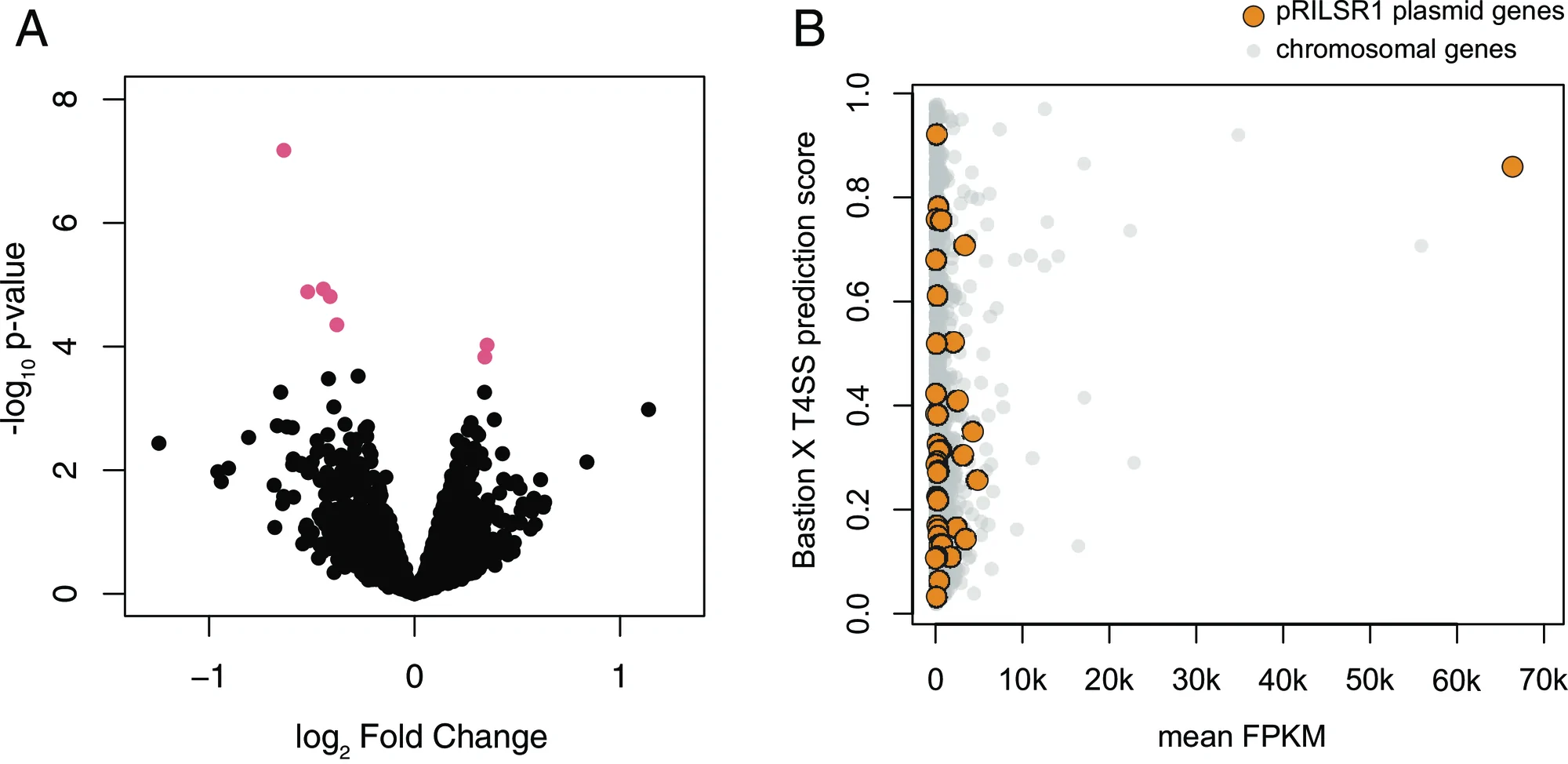
Bacterial gene expression analyses. (*A*) A volcano plot comparing *Regiella* gene expression (both chromosomal and plasmid genes) in *Pandora-*infected vs. uninfected aphids. Each point represents an individual bacterial gene, the x-axis shows the log 2-fold change comparing *Pandora-*infected vs. control libraries, and the y-axis shows statistical significance with genes with an FDR < 0.05 shown in red. (*B*) Shows the results of a computational prediction of T4SS effectors using BastionX, with prediction scores shown on the y-axis. The x-axis shows the strength of expression of each gene represented by FPKM. Orange points indicate pRILSR1 plasmid genes, and gray points indicate chromosomal genes. The highly expressed predicted effector with conserved SifA domain is at the far right of the plot.

Together, these results suggest that the T4SS-encoding pRILSR1 plasmid mediates *Regiella*’s fungal protection phenotype. This is supported by the absence of virD—a critical T4SS component (38, 39)—in the partial plasmid carried by the nonprotective MeliA18 strain. To identify candidate T4SS effectors, we combined BastionX, a secreted-substrate predictor for Gram-negative bacteria (40), with gene-expression data from RNAseq. BastionX predicted numerous putative T4SS substrates on both the pRILSR1 plasmid and the LSR chromosome. RNAseq data from *Pandora*-infected and uninfected aphids containing the protective LSR *Regiella* strain revealed few genes were differentially expressed in response to fungal infection (Fig. 4*A*). However, several candidate T4SS effectors were highly expressed (Fig. 4*B*) in the *Regiella* transcriptome. Notably, the single most highly expressed gene by *Regiella* (BastionX score 0.859; FPKM = 66,386) is a pRILSR1-encoded predicted T4SS effector (NCBI Reference Sequence: WP_039906807.1; this study: LSR_WP_02603). Expression of this gene was nearly 100X higher than the average gene in the *Regiella* transcriptome (Fig. 4*B*). BLASTp analysis identified homologs in several insect symbionts, including *Hamiltonella*, *Arsenophonus*, *Symbiopectobacterium*, and *Fukatsuia*, although all were annotated as hypothetical proteins. InterProScan identified a conserved SifA-like effector domain (G3DSA:3.30.2440.10) in the C-terminal region of the protein. Combined with its high expression, these features support this candidate gene as a protective effector. However, functional characterization will be required to determine the role of this and other pRILSR1-encoded genes in fungal protection.

## Discussion

Facultative symbionts can profoundly shape host biology, yet the mechanisms generating phenotypic variation among natural populations remain poorly understood. Here, we show that gains and losses of a plasmid (called pRILSR1) are associated with divergence in symbiont-mediated fungal protection among sympatric aphid populations. We identified pRILSR1 in distantly related *Regiella* clades associated with pea aphids, consistent with horizontal transfer among strains (36). In laboratory infections, pRILSR1 presence perfectly predicted fungal protection, and plasmid loss during culture eliminated the protective phenotype, linking pRILSR1 to symbiont-mediated defense. Combined with field data showing systematic variation in plasmid frequency and symbiont-conferred protection across host plant-adapted aphid populations, our findings reveal that MGE dynamics contribute to ecologically structured variation in symbiont-conferred defense. More broadly, because adaptive symbiont traits are frequently encoded on MGEs across insect systems (13–19, 41, 42), gain and loss of these elements may represent a general mechanism generating ecologically relevant trait variation among host populations (43).

The pRILSR1 plasmid encodes a complete virB-like T4SS (34). Given the established role of T4SSs in delivering effector proteins into eukaryotic cells (38, 39, 44), we hypothesize that *Regiella* employs this system to secrete factors harmful to *Pandora*, either into the host hemolymph or directly into fungal cells. Effector prediction analysis identified several candidate T4SS effectors on pRILSR1, including a short protein bearing a SifA-like domain. In *Salmonella*, *sif* genes function as Type III secretion system (T3SS) effectors that promote intracellular survival (45). *Regiella* encodes chromosomal T3SSs (34), raising the possibility that this protein is secreted through those systems rather than the pRILSR1-encoded T4SS, which may primarily function in plasmid transfer. Another possibility is that the protein has been co-opted for secretion via a T4SS. Such shifts in secretion-system specificity are known in other bacteria, including YopJ-family effectors that are secreted by a T4SS in *Bartonella* (46). Functional studies, including structural and domain analyses, will be required to determine the secretion route and function of this candidate effector.

Variation in the benefits conferred by heritable symbionts can drive substantial phenotypic differences among host populations (8–11). *Regiella* shows a strong and repeated association with *Trifolium*-feeding pea aphids worldwide (29–31). Surprisingly, our field surveys show that *Regiella* does not protect *Trifolium-*adapted aphids from *Pandora*, indicating that this pattern cannot be explained by variation in fungal defense. Instead, other symbiont-encoded traits are likely contributing to the high frequency of *Regiella* on clover. Early work on the TUt strain reported improved aphid performance on *Trifolium* (28). However, later studies of European *Regiella* strains found limited effect on host plant adaptation (47, 48), pointing to substantial strain-level heterogeneity. Our genomic analyses reveal that TUt harbors a distinct complement of genes lacking known homologs. However, since this strain was sequenced in a previous study, we also cannot exclude the possibility that some genetic elements, such as plasmids, remain uncharacterized. Such elements could encode ecologically relevant functions, including detoxification of plant secondary compounds, enabling rapid, modular shifts in symbiont phenotypes. Clade 2 *Regiella* also attain higher within-host densities and are better competitors than clade 1 strains (49), potentially due to pilin genes encoded on P1 plasmids (50), which may influence their prevalence in natural aphid populations. Future research should focus on determining the genomic origins of diverse *Regiella*-conferred traits, and whether they are encoded on chromosomes or MGEs, as this is key to understanding how heritable symbionts generate the functional diversity that drives host adaptation.

Recent advances in culturing and long-read sequencing are transforming our ability to resolve insect symbiont genomes (41, 51). Our recovery of plasmids absent from previous *Regiella* assemblies illustrates how incomplete mobilome characterization can obscure the functional diversity of symbiont strains. Our data also point to a deeper evolutionary transition. Two *Regiella* strains from *D. aceris* form a highly divergent clade characterized by reduced genome size, low GC content, and an accumulation of ancient transposable elements, which are hallmarks of genome erosion associated with co-obligate symbiosis. In addition, *Regiella* is ubiquitously associated with *D. aceris* aphids (52), suggesting that it may compensate for metabolic functions lost in *Buchnera* in this host, paralleling transitions observed in other aphid systems (53). If confirmed, this would represent a previously unrecognized case of *Regiella* transitioning toward obligate symbiosis, illustrating the dynamic evolutionary trajectories of facultative symbionts.

Strain-level variation often explains more phenotypic variation than bacterial species identity in microbial symbioses, underscoring the limitations of species-level classifications in microbial ecology (54). This principle is likely even more pronounced in complex microbiomes, such as those of the gut, where bacterial genomes are highly dynamic due to frequent gene gain and loss mediated by MGEs. Pangenomes, rather than core genomes, often dictate functionally significant microbial traits, particularly those that influence host–microbe interactions. Our study shows how the dynamics of MGEs contribute to variation even in the comparatively stable context of insect symbiosis. In many insect groups, long-term diversification has been shaped by symbiotic associations, with facultative symbionts contributing to ecological adaptation. Together, our results suggest that MGE mobility contributes to phenotypic divergence among insect populations, with consequences for their responses to ecological pressures.

## Methods

### Sample Collection for Analysis of *Regiella* Genetic Diversity.

We collected aphids in the United Kingdom from 2011 to 2020 from multiple host plants and preserved them in ethanol. We extracted genomic DNA from individual aphid samples using DNeasy Blood and Tissue kits (QIAGEN, Venlo, Netherlands) under recommended conditions. To identify aphid species, we amplified an approximately 700 bp DNA fragment of the cytochrome c oxidase I (COI) “barcoding” gene from the DNA using the Lep_F and Lep_R primers (55), and Sanger sequenced the PCR products in the forward direction. We aligned DNA sequences with MUSCLE (56) and identified aphid species by comparing COI sequences to the BOLD database (http://www.boldsystems.org/) and GenBank using BLASTn (57).

### Screening and *Regiella* Genotyping.

We screened aphid genomic DNA for *Regiella* using diagnostic PCR of the 16S ribosomal RNA gene (58). For samples containing *Regiella*, we used a MLST scheme to characterize *Regiella* strains based on five housekeeping genes: *accD, gyrB, murE, recJ,* and *rpoS* (58). MLST amplicons were Sanger-sequenced in the forward direction. We used Tracy (59) for base calling on the chromatograms to confirm nucleotide substitutions, and we aligned concatenated MLST genes using MUSCLE with default parameters. *Regiella* strains were assigned to a unique sequence type (ST) if they differed by at least one allele. For samples with incomplete data due to PCR amplification failure, STs were defined using the available alleles only. Phylogenetic analyses (see below) were performed on a concatenated MLST alignment and included only STs with data for four or more alleles, yielding 72 unique STs.

### *Regiella* Culturing, DNA Extraction, and Genome Sequencing.

We established in vitro cultures of *Regiella* strains LSR, MD10, MeliA18, 313, T8, T5, and 5.15 in insect cell lines (13). These strains all originated from pea aphids (*A. pisum*) with the exception of strain 5.15, which was isolated from *Myzus persicae* and then transferred into *A. pisum* for lab colony maintenance and initiating the in vitro culture. We attempted to establish strain RR5 (from *Sitobion avenae* transferred into *A. pisum*) in culture, but this produced no visible growth. We extracted DNA from bacterial pellets as described in previous work (60). Purified genomes of *Regiella* were sequenced on a PacBio Sequel II platform at an average yield of 150,000 reads of 10kbp length per sample. Strain LSR was sequenced using an ultralow input sample prep method, giving a yield of 1.4 million reads of 10kbp length. We also performed genome sequencing on 12 additional strains of *Regiella* (Table 1) using gDNA extracted with the EZNA Insect DNA kit (Omega Bio-Tek, Norcross, GA) with an Illumina NovaSeq S4 following the NEBNext Ultra II FS kit library prep protocol to generate paired-end reads of 2 × 150bp length at an average of 200 million reads per sample. These samples were sequenced at the Centre for Genomic Research at the University of Liverpool. A final sample (strain CF7) was sequenced on DNA extracted using the Qiagen DNEasy kit at Novogene, Inc., on an Illumina NovaSeq PE150 platform to 15 Gbp.

### Genome Assembly.

We checked reads for quality and adapter content using FastQC and MultiQC (61) and used FastP (62) with default parameters for trimming. We assembled the PacBio long reads using MetaFLYE v2.9 (63) with parameters --pacbio_hifi and five polishing iterations. We assembled the Illumina reads in two steps. First, we used SPAdes v3.15.4 (64) in --assembly-only mode with default parameters. We then mapped the reads to the assembly using BWA-mem (65) and assigned taxonomic identities to the contigs using megablast (66) and DIAMOND (67) searches against the NCBI’s nonredundant Refseq nucleotide and protein databases, respectively. We used BlobTools v1.1.1 (68) to examine and extract the contigs matching *Regiella* showing distinct coverage and GC content. Then, we extracted *Regiella* reads with SAMtools (69) and reassembled them separately to give more contiguous and complete genomes with SPAdes v3.15.4 (64) in --careful mode using error correction and kmer sizes of 33, 55, 77, 99, and 127.

We manually inspected all final assembly graphs in Bandage (70) for uniform coverage and contiguity, and we performed BlastN (57) searches against the NCBI nonredundant nucleotide database to validate the taxonomic identities of the contigs. We assessed the completeness and contamination levels of the assemblies with CheckM (71) using f_Enterobacteriaceae as the marker lineage that has 546 genomic marker genes. We checked the genomic markers and functional genes that were automatically assigned as missing using HMM (72) and BLAST (57) searches of the target genes on the raw contigs from the whole-aphid initial assembly. To validate plasmid presence in short-read–sequenced strains, we constructed a reference database comprising circularized plasmid sequences from PacBio-sequenced strains, 25 previously described plasmids from *Regiella*, *Hamiltonella*, and *Fukatsuia*, and nucleotide sequences representing all known plasmid incompatibility (Inc) groups. This database was used to query contigs from Illumina-sequenced strains using BLASTn. Because many plasmids share conserved backbone and maintenance genes, we applied a stringent threshold of >80% sequence coverage to robustly infer plasmid presence.

### Phylogenetic Analyses.

We used PhyML (73) with default parameters and 500 bootstraps to reconstruct the *Regiella* MLST phylogeny, and ITOL (74) to visualize the phylogenetic tree. To construct the genome-based phylogeny of all the assembled *Regiella* strains and the previously sequenced TUt strain (GCA_013373955.1), we ran the GToTree v1.8.2 (75) pipeline using HMMER3 v3.3.2 (72), Muscle 5.1.linux64 (56), TrimAL v1.4.rev15 (76), Prodigal v2.6.3 (77), ModelFinder (78), UFBoot2 (79), GNU Parallel v20230922 (80), and IQ-Tree v2.2.5 (81) in default settings with 1,000 bootstraps in RAxML v8 (82) on a concatenated alignment of the 147 orthologous genes universally present in Gammaproteobacteria. We visualized the phylogeny and rooted it to *Fukatsuia symbiotica* strain 5D (GCA_003122425.1) and *H. defensa* strain T5A (GCA_000021705.1) in ITOL (74).

### Pan-Genome Analysis.

We used Prokka v1.14.6 with default settings in--compliant mode to annotate all the assembled genomes (83). We performed a new annotation of the previously sequenced TUt genome to ensure uniformity of the annotations. We used Roary v3.13 (84) with -e -i 90 -s settings to identify core genes common at 90% amino acid identity cutoff in all genomes and accessory genes unique to *Regiella* strains. We visualized the pangenome matrix using Phandango (85). We annotated the genes uniquely present and absent in each *Regiella* clade with COG, KEGG, GO terms, and Pfam domains using eggnog v6.0 (86). We visualized the annotated functional groups with GGplot2 in R v4.3.2. through Rstudio (33206f75, 2023-12-17).

### Comparative Analysis of Gene Function.

We further analyzed the genomes to explore the functional categories that had been gained in each *Regiella* clade. We used Phaster (87) on default parameters to identify and extract bacteriophage sequences from the assemblies. We filtered out incomplete sequences and analyzed the complete bacteriophage sequences using PhageScope (88) to determine their taxonomy and generate genome statistics. We identified transposable elements and analyzed them using Earl Grey (89). We identified the presence of genes coding for the synthesis of amino acids, vitamins, carbon, lipid, and nucleotide metabolism and transporters using KofamKoala (90) and analyzed them in KEGGMapper (91). Last, we visualized the protein coding genes that are on plasmids and have a function other than the transmission or replication of plasmids using Circos plots (92). To validate the absence of genes uniquely missing in clades, we manually checked for these genes in the raw assemblies to ensure reported absences were not due to differences in genome coverage or sequencing technologies used.

### Experimental Fungal Infections.

We transferred 11 *Regiella* strains from three clades into a common pea aphid host genetic background [LSR1-01; (37)] using established protocols and waited at least four generations for the symbiosis to stabilize as in previous studies (93). We infected aphids with *P. neoaphidis* using previously described methods (94). Briefly, we exposed aphids to sporulating fungal cadavers in a PVC tube “infection chamber,” with sporulating cadavers placed above the chamber so spores fall onto the experimental aphids. Sporulating cadavers were rotated among the treatment aphids so that all aphids within an experiment are exposed to each set of sporulating cadavers for an equal period of time as in ref. 95. We then transferred aphids to a fava bean plant (5 to 10 aphids per plant) with a sealed cup cage over the plant, which produces humid conditions necessary for *Pandora* to infect aphids. We kept aphids and plants at 20 °C and 16L:8D for 48 h, after which we transferred aphids to a new plant without parafilm. We recorded whether each aphid survived, died of unknown causes, or produced a sporulating cadaver every 24 h. A repeat infection was conducted 2 y later, using strain 313 and strain LSR in the LSR1-01 background. For the experimental infection assay of 11 *Regiella* strains, we used a *Pandora* isolate collected in Knoxville, TN in 2019 established from a pea aphid collected from *Vicia*. For the RNAseq assay and repeat infection, we used a *Pandora* isolate collected in Chapel Hill, NC in 2025 from *Macrosiphum euphorbiae* feeding on tomato. Both fungal isolates were confirmed to be *P. neoaphidis* using PCR amplification and Sanger sequencing of the ITS gene as in previous work (96). To analyze infection data, we tracked the number of sporulated aphids as a binary outcome per individual and analyzed the data using a binomial generalized linear model with symbiont strain as a fixed effect and a logit link function in R v.4.4.3. The first infection with 11 strains also included replicate as a fixed effect. We checked for overdispersion to confirm that a binomial model was appropriate. We then conducted post hoc pairwise comparisons of each symbiont strain relative to LSR1-01 using the multcomp package (97).

### Nanopore Sequencing of Strain 313 After Loss of Fungal Protection.

We collected hemolymph from aphids and filtered through a 10 μM spin column to enrich for *Regiella*. We extracted DNA using a Qiagen DNeasy kit and conducted library prep using Oxford Nanopore Technologies v.14 ligation kit and the NEB companion module v.2 and sequenced on an R10.4.1 flow cell for 24 h to generate sequence data. We mapped reads to *Regiella* strain 313 (including the pRILSR1 plasmid) using minimap2 (v2.24) (98). Reads that mapped to the genome were extracted for subsequent assembly. Reads (167,580 total reads; 121.1 Mb total sequence) were assembled de novo using Flye v2.9.6 (99) with a genome size estimate of 2.2 Mb and default parameters and polishing using Medaka v2.2.1. We assessed genome completeness using BUSCO v6.0.0 (100) with the enterobacteriaceae_odb12 lineage dataset (prokaryota, 2026-05-22). To assess strain identity, we extracted complete single-copy BUSCO genes from the assembly and aligned them to the reference strain 313 genome using minimap2 (v2.31) with parameters -k15 -w10. Nucleotide identity for each gene was calculated from the alignment edit distance (SAM NM tag) as the number of matching bases divided by the aligned length, expressed as a percentage.

### Field Study Collections and Sample Storage.

We collected adult pea aphids in consecutive springs (2021–2022) in Knox County, TN feeding on species from two genera of host plants in the family Fabaceae (*Trifolium campestre, T. incarnatum, T. pratense, T. repens, Vicia grandiflora, V. sativa, V. villosa*). Aphids were collected from seven sites: Tommy Schumpert Park (160 acres; N 36.046, W -83.958), Cherokee Farms Park (147 acres; GPS: N 35.946, W -83.952), Baker Creek Park (23 acres; N 35.942, W -83.890), I.C. King Park (10 acres; N 35.903, W -83.940), Tyson McGee Park (13 acres; N 35.951, W -83.942), Knoxville Botanical Garden (47 acres; N 35.982, W -83.883), and Henry Ave (5 acres; N 35.941, W -83.912). We visited sites weekly from April 6 through June 21, 2021 (76 d), and in 2022 from March 28 through June 14 (78 d).

To collect aphids, we placed a plastic tray under each host plant and hit the stem with a stick to dislodge them. One adult pea aphid per tray was transferred to a 100 mm Petri dish containing cuttings of the host plant from which it was collected in agar to prevent desiccation. We sealed the dishes with Parafilm and stored them at room temperature for 5 d, after which we scored each aphid as healthy, dead with visible fungal infection (fungal cadaver), dead with visible wasp parasitism (mummified), or dead from unknown causes. We then stored each aphid individually at −20 °C in microcentrifuge tubes; when present, up to five clonal offspring per adult were stored separately.

### Field Sample DNA Extraction and Screening.

We extracted DNA from 926 adult aphids using “Bender buffer” as in ref. 101. In short, we crushed frozen aphids with sterile pestles in microcentrifuge tubes and heat-treated (2 h at 65 °C) each sample with the lysis buffer and Proteinase K (Fisher Bioreagents). We subsequently treated samples with 8 M potassium acetate, vortexed each tube, and kept extractions on ice overnight followed by three ethanol washes and centrifugations. Following precipitation, we eluted the DNA pellet in nuclease-free water and diluted each sample to 20 ng/μL.

We carried out diagnostic PCR screens for seven bacterial symbionts: *R. insecticola*, *H. defensa*, *Serratia symbiotica*, *Spiroplasma* sp., *Rickettsia* sp., *Rickettsiella viridis*, and *Fukatsuia symbiotica*. We used the following PCR program: 94 °C 5 min, 11 cycles of 94 °C 30 s, 56 °C (declining 1 °C each cycle) 50 s, 72 °C 90 s, 25 cycles of 94 °C 30 s, 45 °C 50 s, 72 °C 90 s, and a final extension of 72 °C 5 min as in ref. 32. For each PCR reaction, extracted DNA from known symbiont-containing aphids was used as positive controls, while sterile water was used as the negative control. We extracted DNA from clonal offspring of a subset of samples and screened for symbionts to assess agreement between adult and clone symbiont communities, and we found strong concurrence between PCR reactions from adult fungal cadavers and offspring.

### Strain Typing of Field *Regiella*.

To characterize the genetic diversity of *Regiella* field samples, we used two methods. For the majority of samples, we amplified and sequenced the *murE* housekeeping gene specific to *Regiella* containing samples using a primer pair and PCR recipe as above (32, 58). Amplicons were gel extracted and purified (Zymo Clean and Concentrator Kit under recommended conditions) and sequenced in forward and reverse directions using Sanger sequencing. A representative sample from each clade was used as a PCR positive control as well as for sequencing. Consensus sequences of experimental samples and representative samples (LSR—clade 1; 313—clade 2) were generated by aligning forward and reverse sequences with ends trimmed by hand. A maximum likelihood phylogeny was generated as above to assign strains to each clade. For some samples we amplified a polymorphic region of the *Regiella* genome where some strains have a 32 bp deletion in the amplicon as in ref. 36. Amplicons were visualized on a 3.5% agarose gel run at 70 V on ice for 3.5 h to reduce any gel streaking and confirm the size of amplicons and assign remaining samples to clade 1 or 2. Samples that failed both PCR screens for *Regiella* diversity (n = 6) were included in results after rescreening with original symbiont check PCR and removing false positives.

### PCR Screening for pRILSR1 Plasmid.

We designed primers that amplified a 701 bp region of the pRILSR1 plasmid (133F 5′-CAACGTATCAGCGCCAATGG-3′ and 834R 5′-CGGCTCGATGCTGTTTTCTG-3′). We used NEB 2X Quick Load Master mix to run 20μL reactions that included 10μL of master mix, each primer at 10 μM, and 40 ng of gDNA. PCR conditions included an initial step of 95 °C for 3 m, 40 cycles of 95 °C for 30 s, 56.5 °C for 30 s and 72 °C for 60 s, followed by a final extension step of 72 °C for 5 min and a 12 °C hold. We screened each of the *Regiella* positive samples for the plasmid. We analyzed the percent of *Regiella* from each clade and each host plant with the plasmid and the effect of plasmid carriage on fungal infection using generalized linear models with a binomial error structure, comparing models using Chi squared tests, implemented in R v.4.4.3 (102).

### T4SS Effector Prediction and RNAseq.

For T4SS effector prediction, the *Regiella* strain LSR genome annotation (from this study) was used to extract the protein sequences for each predicted gene using gffread. We uploaded these to Bastion Hub (https://bastionhub.erc.monash.edu/index.jsp) and analyzed sequences using BastionX V2.0 on the highest sensitivity settings. To look for conserved domains in candidate genes, we used Interpro v 106.0, using InterProScan 5.75-106.0. For RNAseq, we collected aphids from the LSR1-01 background harboring strain LSR at 60 h after fungal exposure, and stored samples at −80 °C. We also included control samples at the same timepoint that were handled similarly but not exposed to fungus. We selected 60 h because previous work has shown that the fungus takes between 24 and 48 h to cross the aphid cuticle (103), but by 72 h has converted much of the aphid’s biomass to mycelium as it prepares to produce a sporulating cadaver. We extracted RNA from each sample by macerating whole aphids in groups of three individuals per treatment per timepoint in 300 μL Trizol (Invitrogen; Thermo Fisher Scientific, Inc.) with 100 μL BCP (1-bromo-3-chloropropane; Life Technologies, Thermo Fisher Scientific, Inc.) and standard isopropanol precipitation. We resuspended each RNA pellet in 40 μL nuclease-free water, removed genomic DNA with the Zymo DNase I Reaction Mix according to the manufacturer instructions (Zymo Genetics, Inc.) and cleaned and concentrated with the Zymo RNA Clean & Concentrator kit under recommended conditions. Library preparation of four biological replicates per treatment including ribosomal RNA removal and sequencing was carried out by Novogene Inc. We mapped reads to a combined fasta file containing pea aphid genome, *B. aphidicola* strain LSR genome, and the LSR genome assembly from above using BWA v.0.7.17 (65), and counted reads aligned to the LSR genome with htseq v.2.0.5 (104). We then assessed differential gene expression using edgeR v.3.40.2 (105). We calculated average FPKM across the eight libraries as read count/(gene length in kb × library size).

## Supplementary Material

Appendix 01 (PDF)

Dataset S01 (XLSX)

Dataset S02 (XLSX)

Dataset S03 (XLSX)

Dataset S04 (XLSX)

Dataset S05 (XLSX)

Dataset S06 (XLSX)

Dataset S07 (XLSX)

Dataset S08 (XLSX)

Dataset S09 (XLSX)

Dataset S10 (XLSX)

## Data Availability

Genome assemblies generated in this study were deposited to Genbank under BioProject PRJNA1406521 (106). MLST sequences are available on Zenodo: https://doi.org/10.5281/zenodo.18489145 (107). Raw reads from RNAseq were deposited to the NCBI Sequence Read Archive (SRA) with BioProject number PRJNA1405767 (108). Raw reads from the nanopore sequencing of strain 313 were deposited to the SRA with BioProject number PRJNA1478964 (109). Raw data from the field experiment are available in Dataset S5, and data from the experimental fungal infections can be found in Dataset S10.
