# Supplementary material for: Phenotypic divergence is driven by mobile genetic elements in a heritable insect symbiont
Source: Proc Natl Acad Sci U S A. 2026 Sep 21;123(39):e2607226123. doi: 10.1073/pnas.2607226123 (PMC13624595; doi:10.1073/pnas.2607226123)
Supplement: Supplementary file 1 [file pnas.2607226123.sapp.pdf]

Supporting Information for: Phenotypic divergence is driven by mobile genetic elements in a heritable insect symbiont

Balig Panossian, Matthew R. Kolp, Taoping Wu, Keertana Tallapragada, Vilas Patel, Elliott B. Goldstein, Kerry M. Oliver, Lee M. Henry,* & Benjamin J. Parker *

* co-corresponding authors: Lee Henry: l.henry@qmul.ac.uk & Benjamin Parker: bpar@unc.edu

This PDF file includes:

Supporting text
Figures S1 to S9
Legends for Datasets S1 to S10
SI References

Other supporting materials for this manuscript include the following:

Datasets S1 to S10

Supporting Information Text

Supplementary Results A: A potentially co-obligate symbiosis in *Drepanosiphum* aphids
Regiella clade 5 represented a highly diverged lineage associated with *Drepanosiphum* aphids, which potentially has evolved to be a co-obligate symbiont along with *Buchnera.* In the *Drepanosiphum* samples we sequenced, the primary nutritional symbiont *Buchnera* has lost the capacity to synthesize histidine, riboflavin, biotin, and thiamine, which are metabolic capabilities retained by the *Buchnera* strains of most other aphids. Additionally, we found that *Regiella* strains from *Drepanosiphum* aphids have retained the ability to synthesize the necessary B vitamins that the primary nutritional symbiont *Buchnera* cannot produce, thereby suggesting that *Regiella* could rescue the nutritional auxotrophies of *Buchnera*. Additionally, *Regiella* from *Drepanosiphum* aphids carried two unique ABC transporters *cydC* and *wzt*, and *ptsO* which is a phosphorylation site that can mediate carbohydrate metabolism. These strains were also missing importers for potassium (*kup*) and thiamine (*thiQ*), and transporters for monovalent cations (*ybaL*), zinc (*zitB*), arginine (*tatC*), glutathione (*kefG*), and *mrp* which can bind and transfer iron-sulphur clusters.

Supplementary Results B: Functional characterization of *Regiella* genomes
Membrane modification genes, transporters, and transcription regulators unique to divergent clades: Certain functional groups of chromosomally encoded genes were differentially present across *Regiella* clades, including membrane modification genes, substrate transporters, and transcriptional regulators. We further explored each of these categories to better understand the gain and loss of genes, and their functions, over *Regiella*'s evolutionary history.

We found a diverse set of genes coding for proteins that alter the bacterial cell envelope to be uniquely present in the strains of each phylogenetic clade. Members of clade 1 had a membrane associated flagellar hook coded by the *filD* gene, clade 2 strains carried a lysyltransferase, *spaS* coding for the surface presentation of antigens, and *ompX* which modifies the bacterial outer membrane proteins, clade 3 strains had genes that attach sugars to the membrane such as a nucleoside-diphosphate-sugar pyrophosphorylase and ADP-glyceromanno-heptose 6-epimerase (*rmd*) and an amidase *amiD*, while clade 4 strains had a membrane peptidoglycan binding lysin motif *nlpD* and flagellar basal body formation genes *flgF/A/G.*

We also identified genes encoding intracellular transporters having differential presence/absence patterns across the phylogenetic clades. Strains of clade 2 *Regiella* contained genes for glycosyl transferases of family type 2 and 8, as well as a queuosine (Q) importer, strains of clade 3 contained genes for the transport of phosphatidylinositol, and a *codB* permease for cytosine, uracil, thiamine, and allantoin, while clade 4 *Regiella* contained genes encoding transporters for 4-amino-4-deoxy-L-arabinose, glycosyl transferases of family type 2 and 8, and the gene *wzt* which functions as an ATP-ase for the ABC-type polysaccharide polyol phosphate transport system.

We found various DNA binding genes that dictate the transcriptional response to external stimuli with contrasting presence/absence patterns between strains of *Regiella* from each of the different phylogenetic clades. Clade 2 *Regiella* strains carried a number of unique transcription regulators including some that could have originated in mobile genetic elements that have

integrated into the chromosome such as *vagC* for plasmid expression control and *cro* for phage-related virulence regulation, as well as global switches that modulate bacterial transcription like *expR* and *gntR*. Interestingly, strains of clade 3 have adopted the osmotically inducible gene *osmE* while *Regiella* from clade 4 use *osmY* to sense osmotic pressure and regulate bacterial growth (Dataset S4).

Supplementary Results C: Additional analysis of mobile genetic elements across the *Regiella* pangenome.

Plasmids: The P1 plasmid contains 8 genes coding for the T4b secretion system *dotB*, *dotC*(x2), *dotD*, and *virD4* (x4), 6 pilin genes *pilO*(x2), *pilV*, *pilT*, *pilS*, and *fimV*, two copies of the thermonuclease gene *nuc*, a gene for the uptake and processing of Queuosine, and a glyoxalase gene. A second (~40kb) plasmid (P2) was unique to strain 5.15 carrying Type 4 secretion system genes *virB8* and *lvhB4*, adhesin gene *yejO*, secreted virulence factor *sopD*, fructose-1,6-bisphosphatase gene Fbp, peptidoglycan modification gene *slt*, and a virulence regulator *cpdA*. A large (~80kb) P3 plasmid was also found to be restricted to members of a unique clade (strains RR5, 5.15, RU4) which carries 8 genes to make the Type 2 secretion system *pilB, pilR, pilS, pilV* (x2), *gspO* (x2), and *pilO*, the gene *str* involved in resistance to antibiotics in the Aminoglycoside family, two copies of the transcription regulator *lysR*, and two copies of the gene *lagB* involved in eukaryotic cell invasion and virulence. The full annotations of the plasmids are available in Dataset S6.

Transposable elements: We found 471 transposable elements (TEs) in the genomes that we collapsed to 66 types based on orthology between TEs found in multiple strains of *Regiella*. A large number of TEs were shared across all the strains, but certain types of TEs were only found in strains of particular phylogenetic clades (Figure S5). Despite the relatively consistent distribution of TEs across the genomes, we found that *Regiella* strains from *Drepanosiphum* aphids have a larger proportion of their genomes covered by TEs (Figure S6). This was not due to an increased number of TEs in *Regiella* from *Drepanosiphum* aphids, but rather due to the smaller genome size of those particular strains of *Regiella.* Additionally, the TE landscape which gauges the recency of TE activity by measuring the genetic distance between TEs of each family has a peak at zero for most *Regiella* genomes except those from *Drepanosiphum* (Figure S6). This suggests that the majority of TEs in *Regiella* genomes have been recently acquired, however, *Regiella* from *Drepanosiphum* aphids do not show evidence of recent TE acquisitions indicating these *Regiella* lineages may no longer be taking up transposable elements.

Bacteriophages: Phages were also a major source of genetic variation among *Regiella* clades. We compared complete bacteriophage sequences to determine their functional gene content and presence in different *Regiella* (Figure S7, Dataset S7). All 20 bacteriophages were taxonomically assigned to the class *Caudoviricetes* (Dataset S2). Interestingly, we found phage diversity had a strong phylogenetic signature across symbiont clades, suggesting several ancestral acquisitions of phage that have co-diversified with *Regiella.* The *Regiella* strains associated with pea aphids on *Trifolium* contain multiple diverse bacteriophages that are only shared by strains from *Metopolophium* aphids within their phylogenetic clade. In contrast,

strains associated with *Medicago* pea aphids carry multiple phages that are exclusively shared by strains from *Macrosiphum* aphids within their phylogenetic clade.

We further found that some of the phages carry bacterial genes that could play a role in expanding the metabolic capacities of *Regiella* strains. Phages found only in the clade 1 strains of *Medicago* pea aphids and *Macrosiphum* carry genes such as suhB and spaA/R involved in modulating Type 3 secretion systems. In addition, another phage from clade 1 *Regiella* contains the genes qmcA and bamE which influence the bacterial outer membrane structure, and atzF, which is involved in the metabolism of *s*-triazine ring compounds. The phages carried by *Regiella* strains from *Trifolium* pea aphids (clade 2) contained the genes AraC a modulator of Arabinose sugar metabolism, UbiC a ubiquinone biosynthesis gene, and the cold-shock protein gene cspE. A large ~110kb bacteriophage (phage 18) contains three genes: folD which modulates tetrahydrofolate interconversion and is a key player in one-carbon metabolism, modA a molybdate uptake protein, and pgl a key enzyme of the pentose phosphate pathway.

Supplementary Results D: Statistical analysis of fungal infection data

We analyzed the experimental fungal infection of 11 *Regiella* strains in the same host genetic background using a binomial generalized linear model with strain as a fixed effect. Post hoc pairwise comparisons were conducted relative to LSR1-01. In the initial infection experiment, four *Regiella* strains significantly reduced sporulation relative to the LSR1-01 control strain: Strain 313 ($z = -3.552$, $p = 0.004$), MD10 ($z = -3.306$, $p = 0.010$), LSR ($z = -3.098$, $p = 0.020$), and T5 ($z = -3.338$, $p = 0.009$). Strains 126 ($z = -1.000$, $p = 0.972$), 42 ($z = -0.551$, $p = 1.000$), 5.15 ($z = -1.376$, $p = 0.817$), CF7 ($z = 0.092$, $p = 1.000$), MeliA18 ($z = -0.420$, $p = 1.000$), P73G ($z = -0.964$, $p = 0.979$), and RR5 ($z = -2.194$, $p = 0.240$) did not significantly differ from LSR1-01.

We repeated this analysis for the second infection after strain 313 plasmid loss. Strain LSR again significantly reduced sporulation relative to LSR1-01 ($z = 4.195$, $p < 0.001$), but we detected no significant difference in sporulation between symbiont-free aphids and those harboring plasmid-free strain 313 ($z = -1.153$, $p = 0.419$).

Supplementary Results E: Nanopore sequencing of strain 313 after protection loss

167,580 total reads mapped to the Regiella strain 313 reference genome, which includes the pRILSR1 plasmid, but no reads mapped to the plasmid itself. The polished assembly of these mapped reads generated 83 contigs with a total length of 2,508,426 bp. The N50 contig length was 155,169 bp, with the largest contig spanning 549,583 bp. Mean coverage across the assembly was 34x. We assessed genome completeness and strain identity using BUSCO v6.0.0 with the enterobacteriaceae_odb12 lineage dataset. Completeness was 77.9% (681 of 874 genes complete, 1.7% fragmented, 20.4% missing). To assess strain identity, complete single-copy BUSCO genes from the polished assembly were aligned to reference strain 313 using minimap2, and nucleotide identity was calculated per gene from the alignment edit distance. Of 677 single-copy BUSCO genes, 668 were identical to the reference and the remainder differed by a small number of positions, with a mean per-gene nucleotide identity of 99.998%, confirming strain identity.

Supplementary Results F: Additional analysis of field data

Statistical analysis of Pandora infection: The Knoxville, TN, USA pea aphid population that we sampled between 2021 and 2022 mainly harbored *Regiella* (25.2%), *Serratia symbiotica* (43.6%), and *Rickettsia* (24.4%), with low or zero frequencies of *Hamiltonella defensa* (1.5%), *Fukatsuia symbiotica* (0.65%), Rickettsiella (1.1%), and *Spiroplasma* (0%) (Figure S8). We analyzed *Pandora* infection among field data using binomial generalized linear models, including pea aphid biotype (*Vicia* or *Trifolium*), presence and absence of *Regiella*, *Serratia*, and *Rickettsia*, and the interaction of each symbiont with biotype. We determined significance by comparing models using ANOVA and chi-square tests. By itself, biotype had no significant effect on the likelihood of *Pandora* infection ($\chi^2 = 0.89$, 924 df, $p = 0.35$). While *Regiella* had no effect on infection ($\chi^2 = 0.62$, 923 df, $p = 0.43$), the presence of *Serratia* and *Rickettsia* ($\chi^2 = 8.8$, 922 df, $p = 0.003$ and $\chi^2 = 10.5$, 921 df, $p = 0.001$, respectively) decreased the likelihood of *Pandora*, likely driven by the documented protective effect of *Rickettsia* [1] and the strong co-infection between these two microbes (see below). However, the protective effect of symbionts on fungal infection varied between biotypes for *Regiella* ($\chi^2 = 3.4$, 920 df, $p = 0.065$) but not for *Serratia* or *Rickettsia* ($\chi^2 = 0.28$, 919 df, $p = 0.6$ and $\chi^2 = 0.005$, 918 df, $p = 0.94$, respectively).

To test whether the protective effect of *Regiella* depended on pRILSR1 rather than *Regiella* presence alone, we compared a model including biotype and *Regiella* presence/absence with a model in which *Regiella* infections were partitioned into three categories: no *Regiella*, *Regiella* without pRILSR1, and *Regiella* with pRILSR1. Twelve *Regiella*-positive aphids with unknown plasmid status were excluded, leaving 914 aphids for this analysis. Incorporating plasmid status significantly improved model fit (likelihood ratio test: $\chi^2 = 6.21$, 1 df, $p = 0.013$), indicating that plasmid carriage explains variation in fungal protection beyond host plant and *Regiella* presence alone.

Patterns of symbiont infections and co-infections in field data: To test if symbiont co-infections were common or rare based on their observed counts, we performed a chi-square goodness of fit test in R version 4.0.3. We found that *Regiella* is more often found in single infections than would be expected by chance (163 single infections vs 96 expected, $\chi^2 = 20.2$, $p < 0.0001$), while *Serratia* and *Rickettsia* were less often in single infections than expected (*Serratia* 174 single infections vs. 221 expected, $\chi^2 = 7.1$, $p = 0.0076$; *Rickettsia* 27 single infections vs. 92 expected, $\chi^2 = 37.9$, $p < 0.0001$). This was driven by an enrichment of *Serratia* and *Rickettsia* co-infections (167 co-infections vs. 71 expected, $\chi^2 = 44.4$, $p < 0.0001$) and a lack of co-infections between *Serratia* and *Regiella* (36 observed vs 74 expected, $\chi^2 = 11.5$, $p = 0.0007$). The complete results of this analysis can be found in Dataset S8. Figure S9 shows the frequency of symbionts and natural enemies over the course of the growing season. Both parasitoid wasps and *Pandora* were important sources of mortality for aphids across the field collections, with a peak of *Pandora* infection prevalence in late spring (Figure S9).

Figure S1

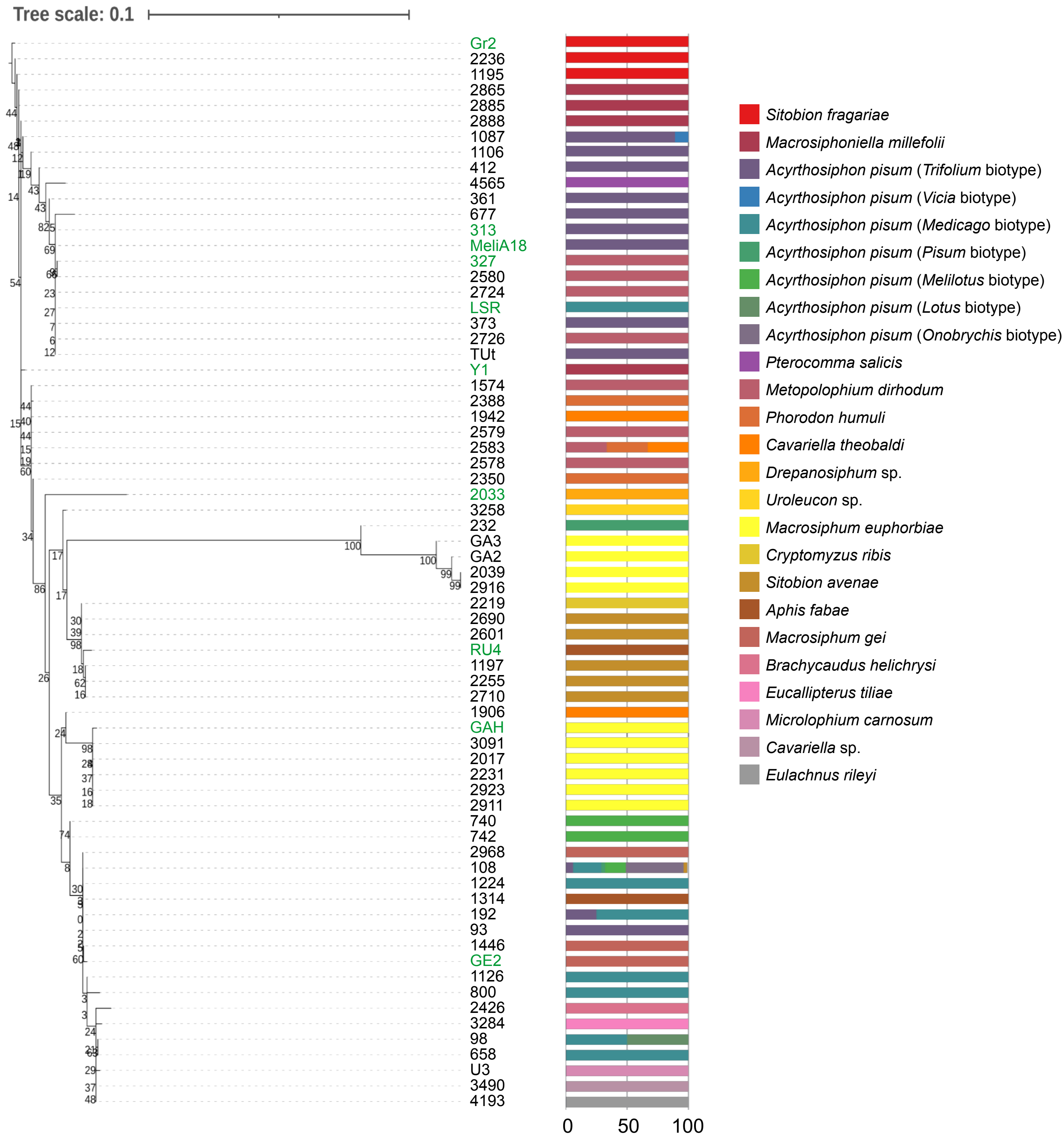

Figure S1: Maximum-likelihood phylogeny of 72 *Regiella* sequence types based on five MLST genes. Bars next to each node indicate the proportions of the aphid host species of the symbionts, as indicated. Tips labelled in green font represent strains used for whole genome sequencing. See Dataset S1 for complete sampling list.

Figure S2

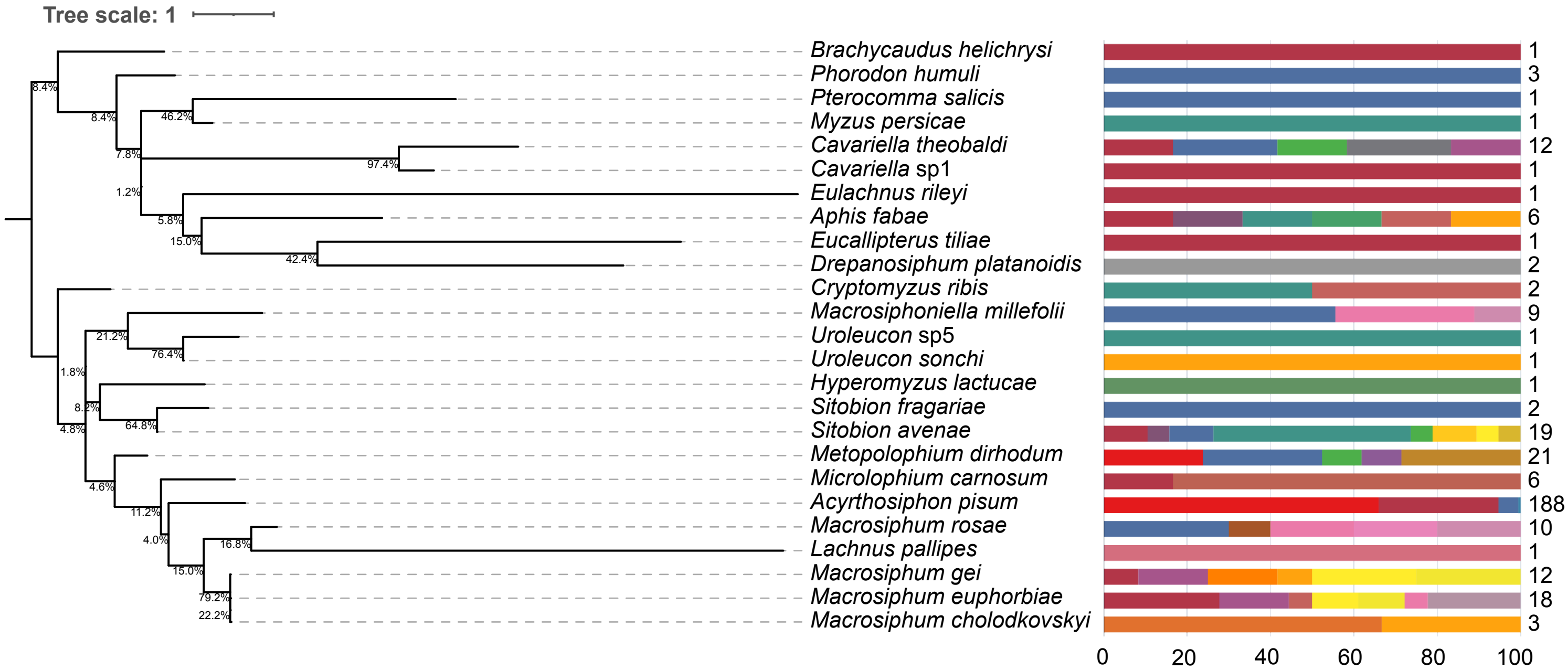

Figure S2: Maximum-likelihood phylogeny of the Cytochrome Oxidase 1 gene copies of the *Regiella*-positive aphids sampled in this study. Bars next to each node indicate the genotype of the *Regiella* strains associated with each aphid strain. *Regiella* strains with more than 99% nucleotide identity were collapsed to single genotypes.

Figure S3

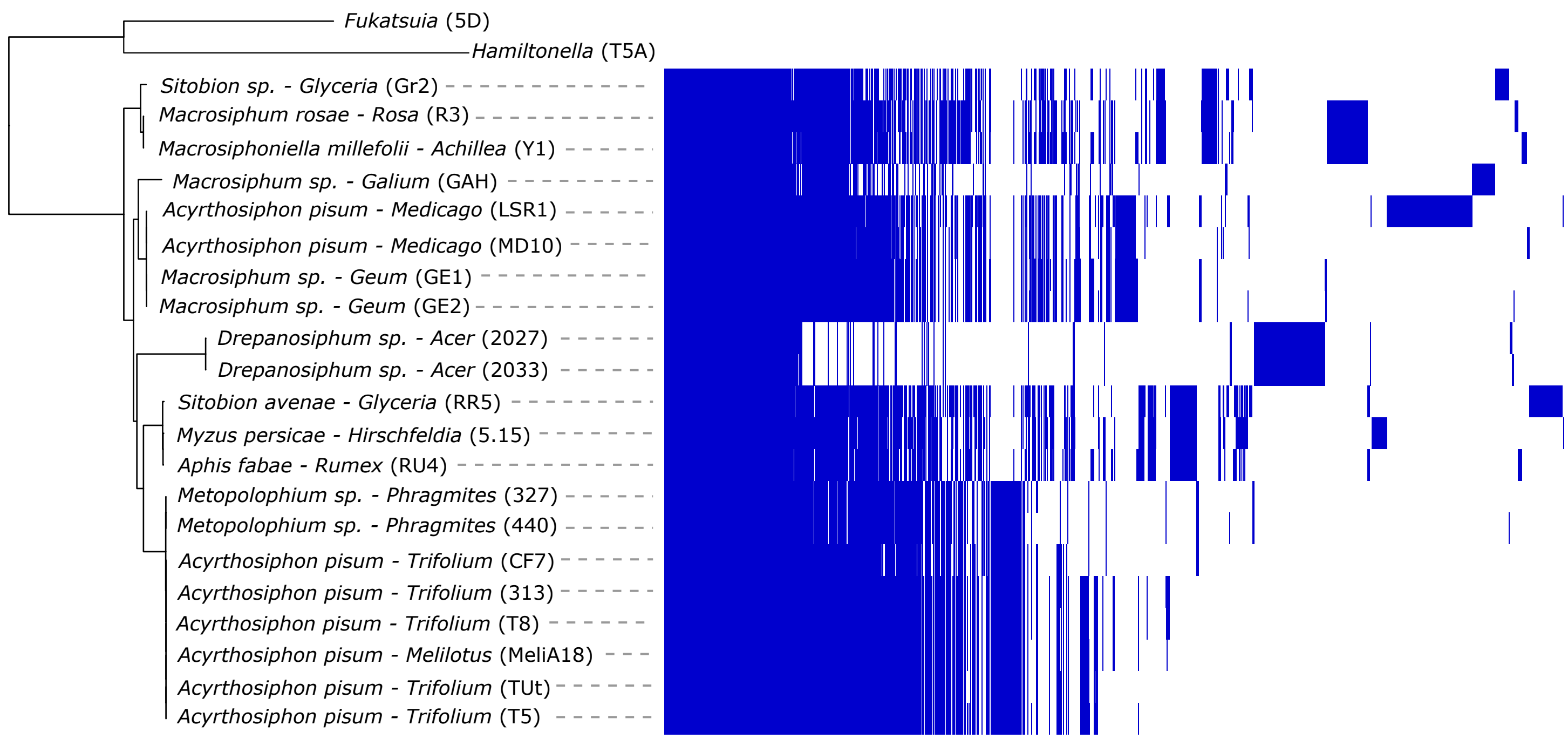

Figure S3: Phylogeny and pangenome matrix of all *Regiella* genomes, visualizing shared genes across strains. The whole-genome phylogeny based on Figure 1 is shown to the left of the figure.

Figure S4

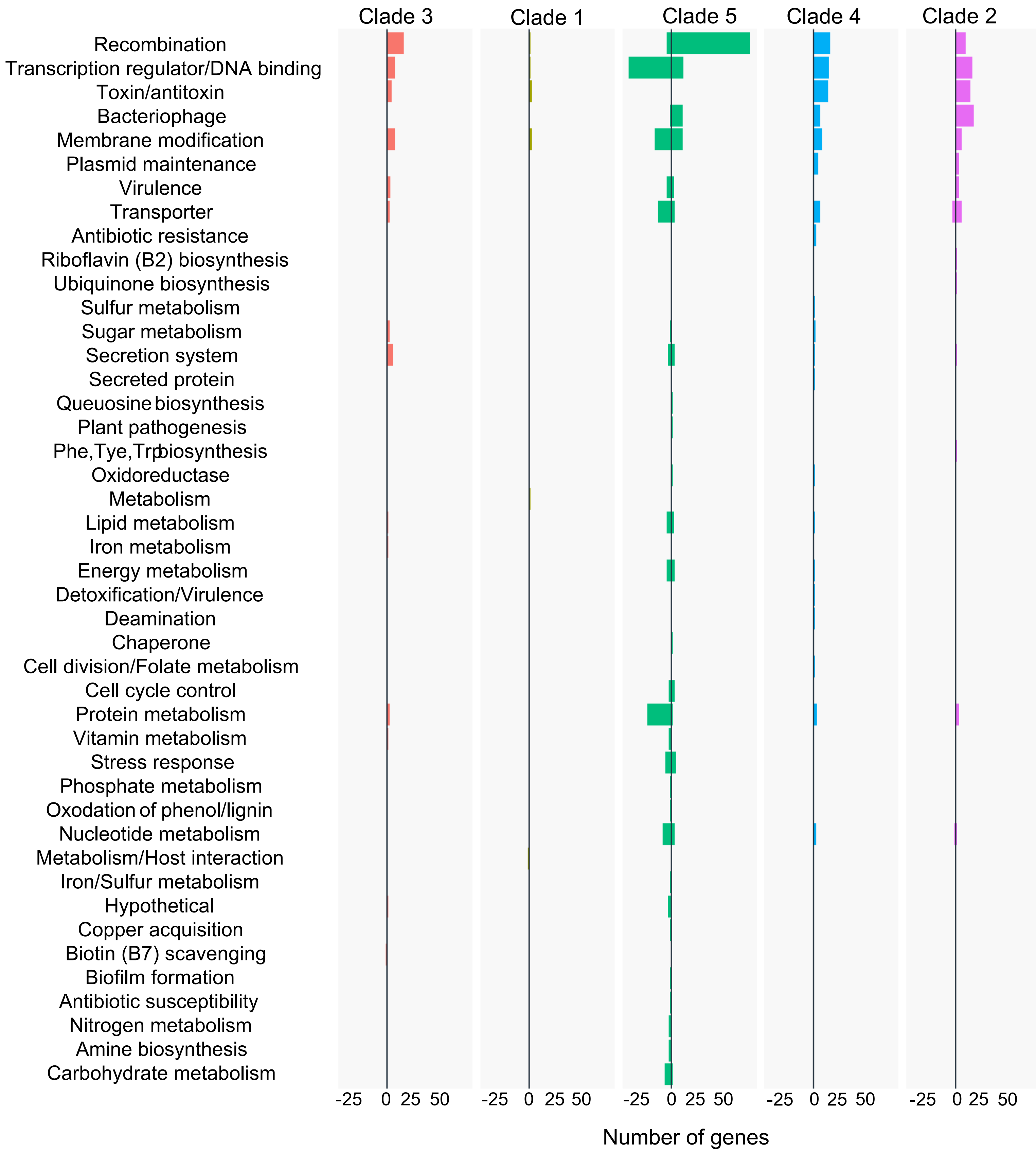

Figure S4: Counts of functional categories of annotated genes uniquely present/absent in each clade of *Regiella*. Functional categories are based on the manual curation of aggregated annotation results from multiple tools GO, COG, KEGG, and Pfam.

Figure S5

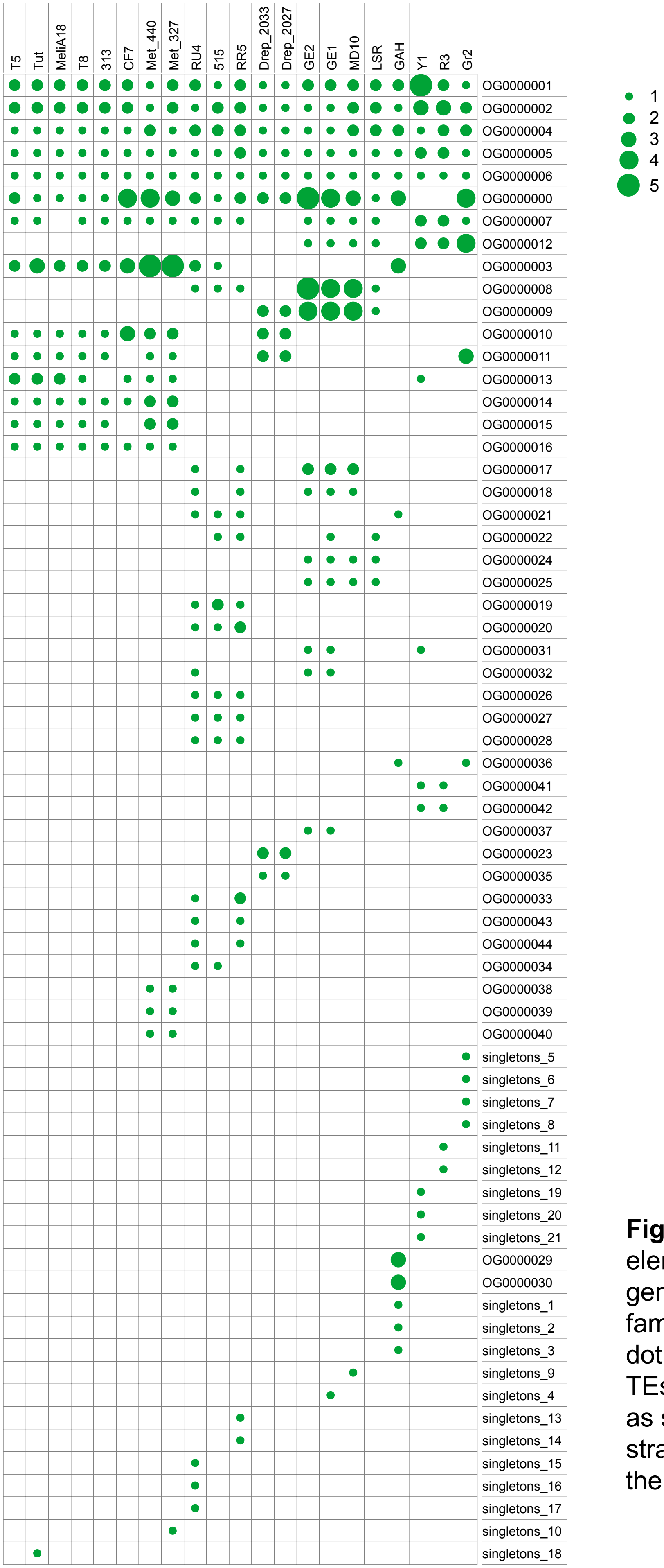

Figure S5: Matrix of transposable elements (TEs) in *Regiella* genomes. TEs are grouped into families as orthogroups (right) and dot sizes indicate the number of TEs per strain in each orthogroup as shown in the key. *Regiella* strains are shown along the top of the figure.

Figure S6

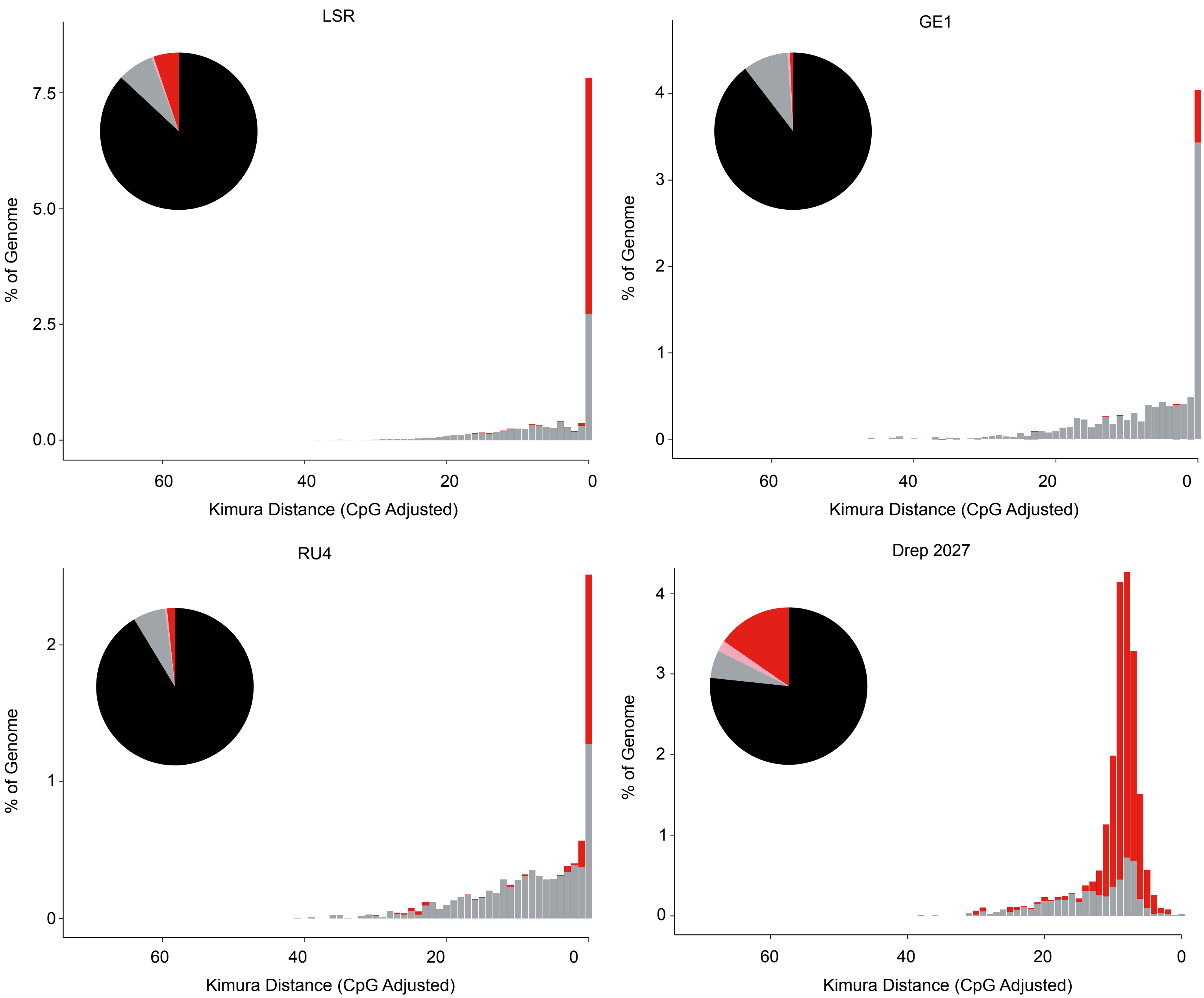

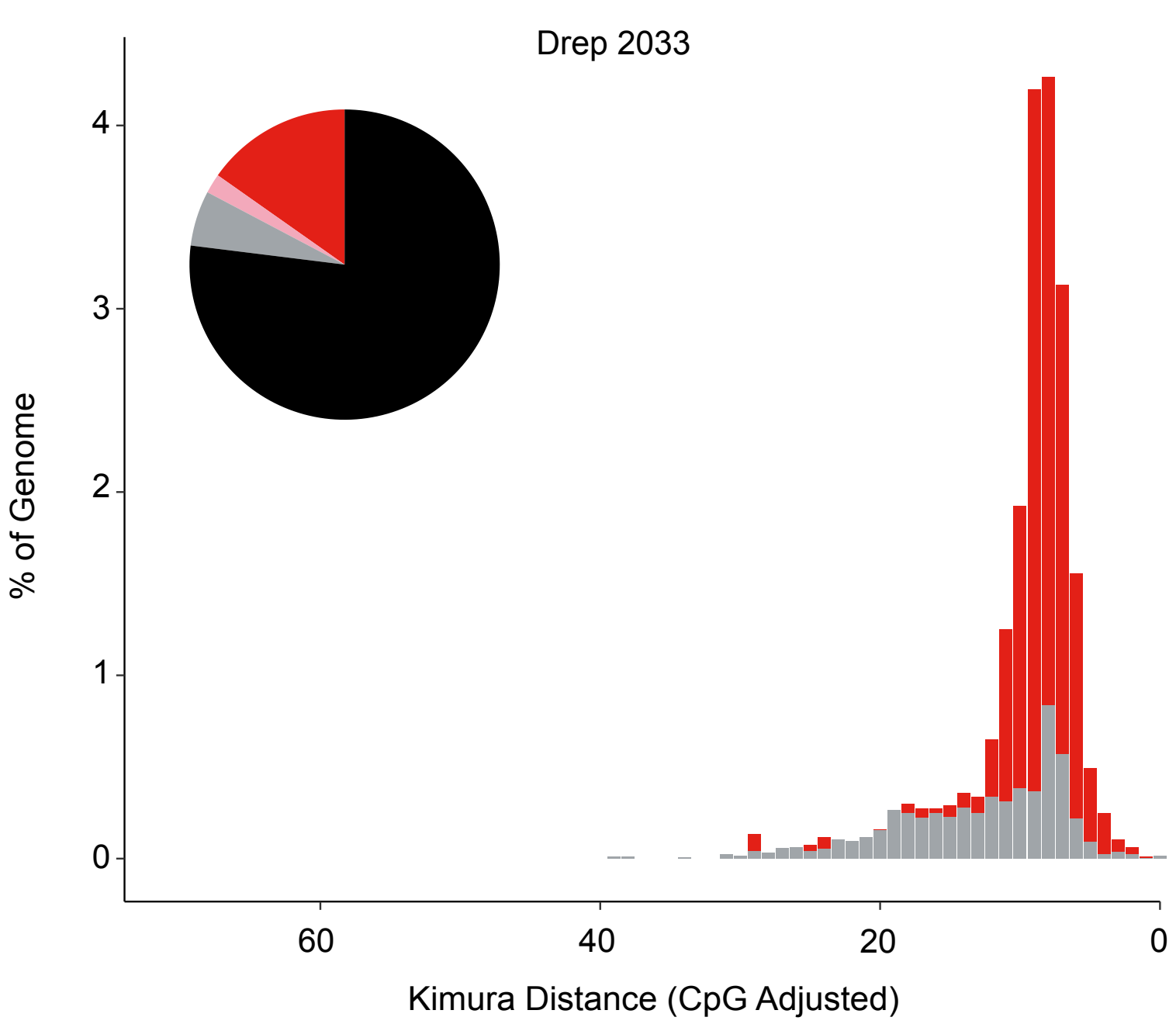

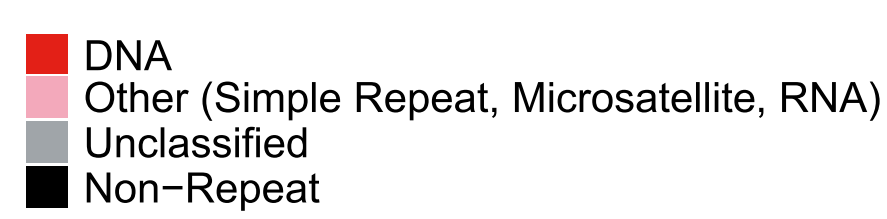

Figure S6: Repeat landscape plots of TEs for five *Regiella* strains. Y-axes show the percent abundance of each TE family, X-axis shows the kimura distance between TEs of each family used as a proxy for relative time of activity in the genome. Pie chart inserts show the proportion of genomes covered by TEs.

Figure S7

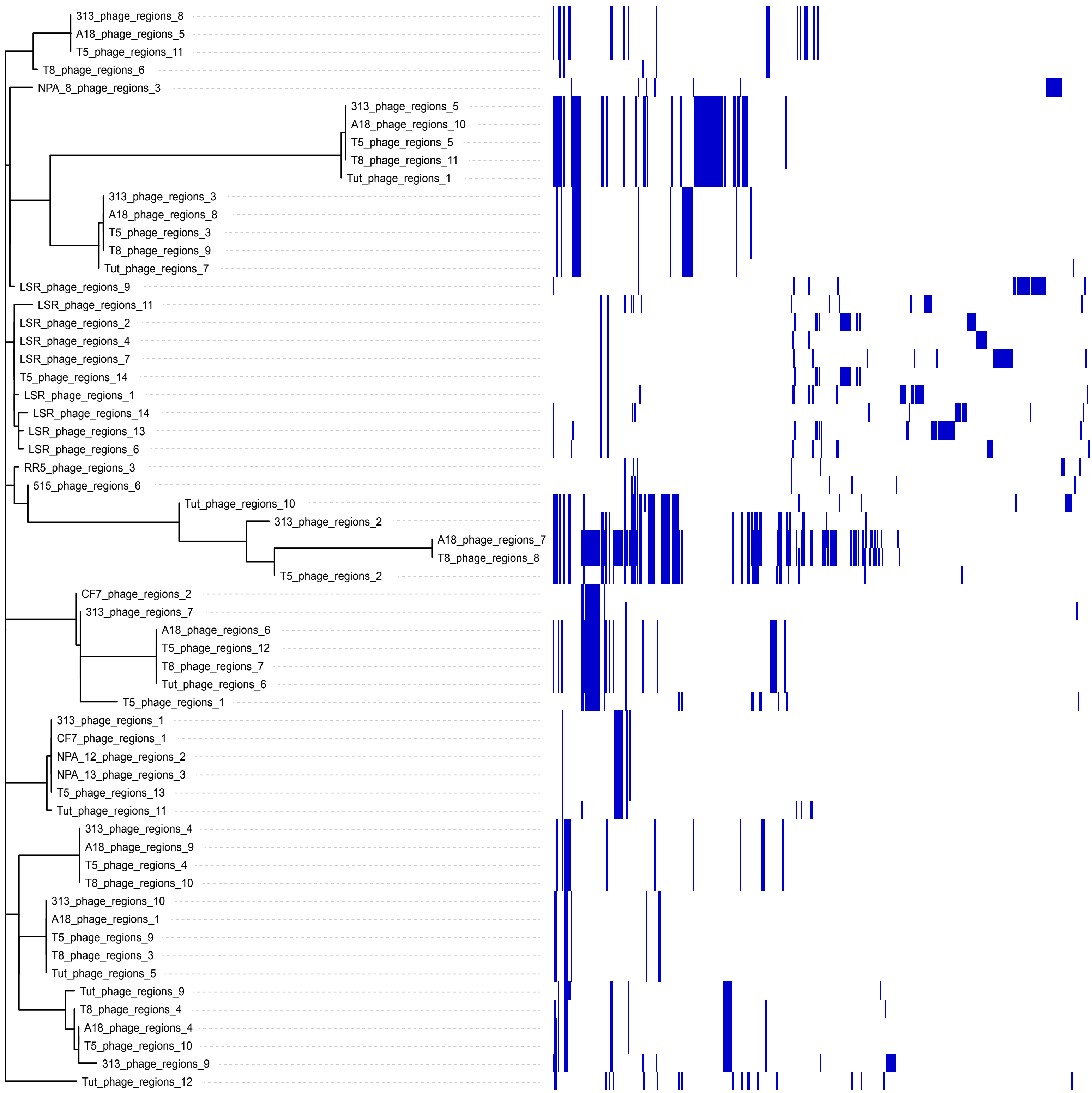

Figure S7: Pangenome matrix of all complete bacteriophages present in the *Regiella* genomes. Phylogeny indicates the relatedness of phages from different strains of *Regiella* based on the number of phage genes shared.

Figure S8

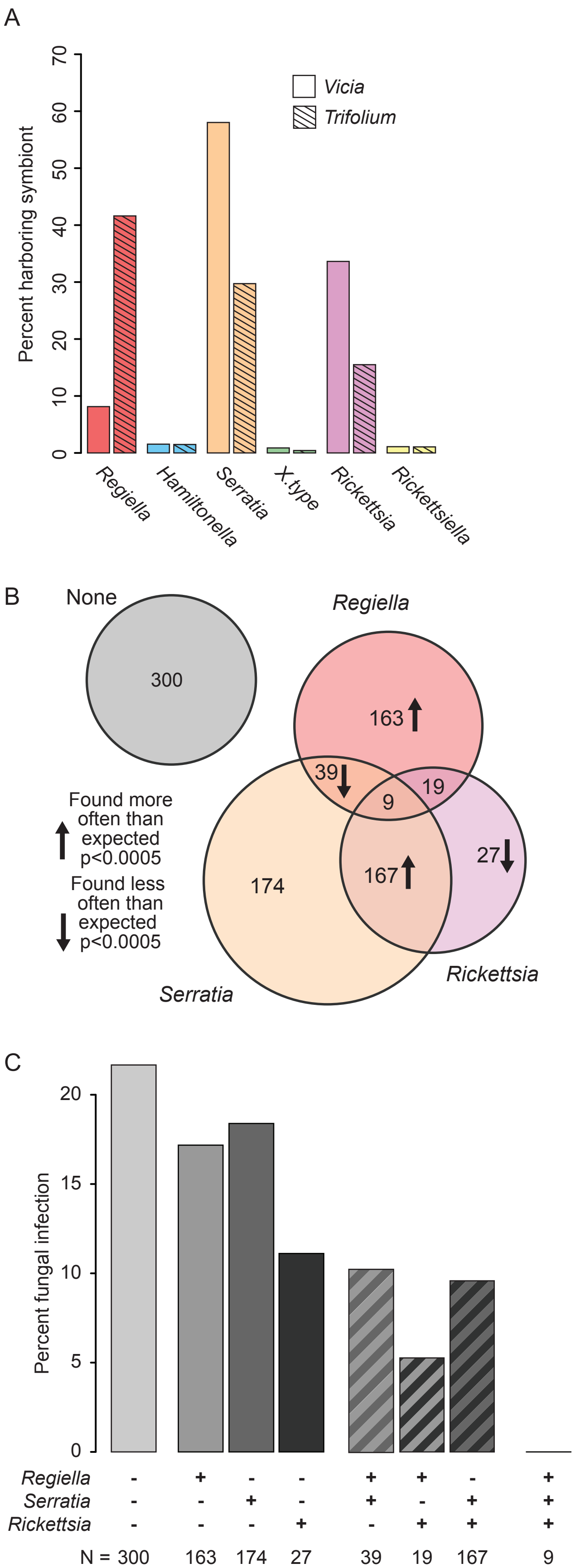

Figure S8: Symbiont infection frequencies and patterns of co-infection based on field data for adult pea aphids collected in Knox County, TN, USA during consecutive springs in 2021 and 2022 feeding on host plants (*Trifolium* spp. or *Vicia* spp.). **A** shows the percentages of aphids collected from either *Vicia* or *Trifolium* harboring a particular symbiont as shown along the bottom of the figure. **B** shows a Venn diagram with co-infection counts for the three most commonly found symbionts: *Regiella, Serratia,* and *Rickettsia*. Arrows indicate significant co-occurrences based on a null Chi-square distribution of symbiont groupings based on random chance with an adjusted alpha of 0.0005. **C** shows the percentage of aphids that died of a fungal infection based on whether they harbored one, two, or all three of *Regiella, Serratia,* and *Rickettsia*, as indicated.

Figure S9

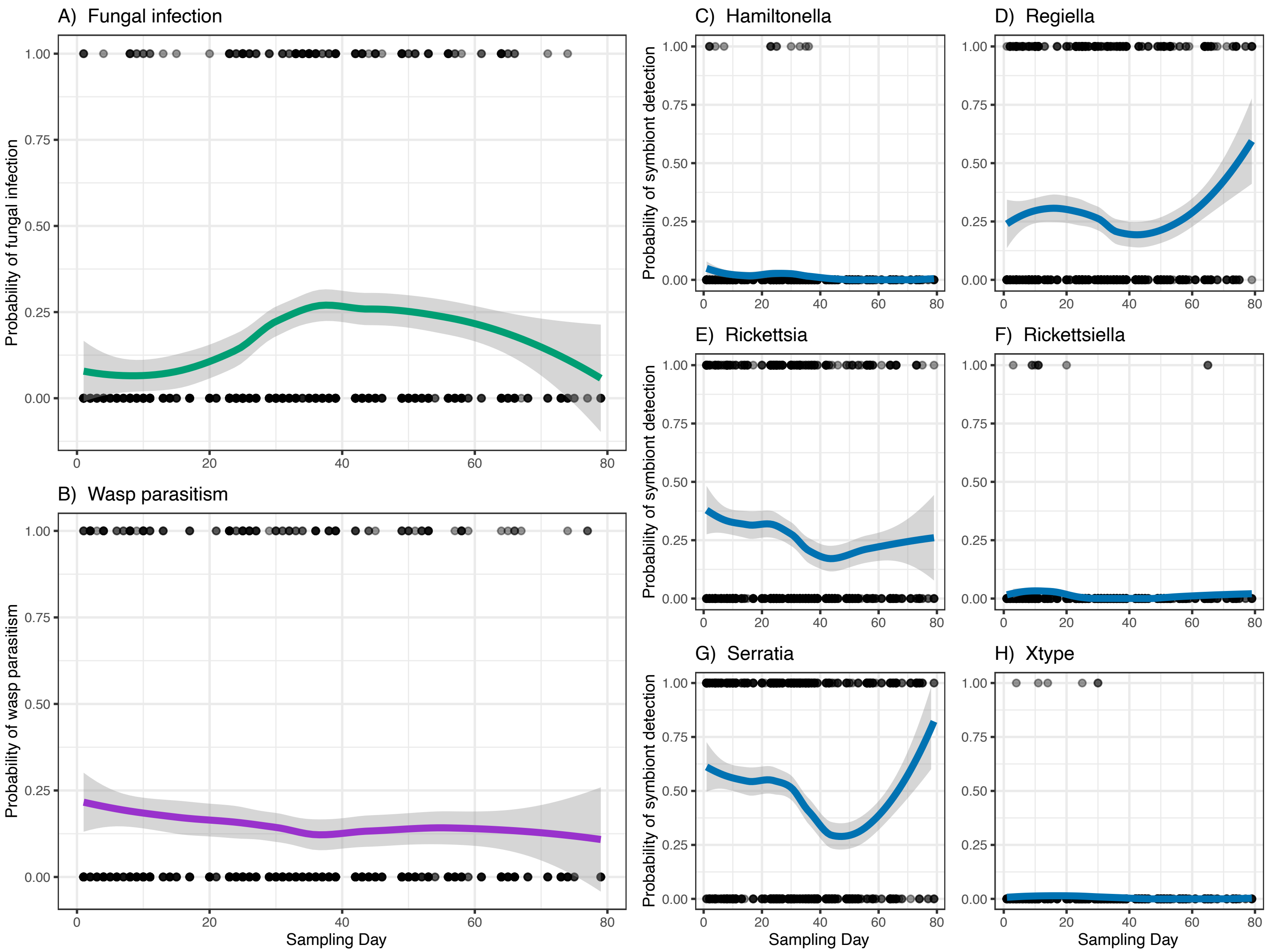

Figure S9: Temporal patterns in the field data. Trend lines were modeled using ANCOVA and the Locally Estimated Scatterplot Smoothing (LOESS) method in ggplot2. Sampling Day refers to the cumulative days after the first collecting day of the year. Panels A and B correspond to the probabilities for fungal infection and wasp parasitism resulting in mummification, respectively. Panels C through H correspond to the probability of detecting a particular symbiont in an individual aphid across the collecting season (note zero *Spiroplasma* symbionts were detected).

Supporting Dataset Captions

Dataset S1: Metadata of collected *Regiella*-positive aphids, aphid genus, host plant, and sequence type (ST) of *Regiella* strains based on 5 MLST genes. Samples selected for genome sequencing and analysis are shaded to match the *Regiella* clades in Figure 1.

Dataset S2: Complete bacteriophages identified in *Regiella* genomes, GC content, phage size, predicted host, lifestyle, and taxonomic classification.

Dataset S3: Pangenome comparison of all genes found in all analyzed genomes of *Regiella* strains. Rows indicate the different annotation assignments for each gene tagged with their respective common gene name, description of the gene product, COG category, GO annotation, EC identifier, KEGG KO number annotation, and Pfam domain identifier.

Dataset S4: Annotations of genes uniquely present and absent in each phylogenetic clade of *Regiella*.

Dataset S5: Data from the Tennessee pea aphid collection experiment. Columns include information from each aphid collected and surveyed, including collection year (Year_id), sample number (Sample_id), the date collected, collection day (with March 28th set as day 0), host plant genus, whether the aphid was winged (as indicated by a 1) or unwinged, whether the aphid died from a cadaver, parasitoid wasp (Mummy), or other source or mortality, the results of PCR assays for *Regiella* along with which clade a positive *Regiella* strain came from, PCR results for *Hamiltonella, Serratia, Fukatsuia, Rickettsia, and Rickettsiella*, a categorical variable describing the complement of symbionts for each aphid, and PCR results for the pRILSR1 plasmid. Note that no *Spiroplasma* strains were detected in our field collections and this column was omitted.

Dataset S6: Annotations of protein coding genes in all 4 plasmids found in *Regiella* genomes, with genes from each plasmid in a separate sheet. Columns indicate the coding regions, common names of predicted proteins, text descriptions, and functional assignments based on COG, KEGG, and PFAM database matches.

Dataset S7: Annotations of protein coding genes in all 20 complete bacteriophages found in *Regiella* genomes. Columns indicate the coding regions, common names of predicted proteins, text descriptions, and functional assignments based on COG, GO, EC, KEGG, and PFAM database matches

Dataset S8: Statistical analysis of symbiont co-infection in aphid populations. Observed versus expected frequencies for solitary and co-occurring secondary symbionts.

Dataset S9: Results of the RNAseq analysis. Each row provides information about an individual gene in the strain LSR genome from the RNAseq study. logFC gives the log 2 fold change comparing control vs. fungal-infected hosts. logCPM is the average read counts per million across the 8 libraries. The F and PValue columns give the results of statistical analysis looking

at differential expression of each gene between control and fungus-infected hosts. Length_bp gives the length in base pairs for each gene, and mean_FPKM is the Fragments Per Kilobase of transcript per Million mapped reads. Bastion_X_T4SS gives the results of the BastionX analysis. Note that genes encoded on the pRILSR1 plasmid have gene IDs LSR_WP_02579 through LSR_WP_02626.

Dataset S10: Results of the experimental *Pandora* infections. The tab labeled “Figure 2A” includes raw data from infection data across 11 *Regiella* isolates in a common host genetic background. Experimental replicate, strain, a binomial term for whether a line contains *Regiella* or not, the initial number of aphids on each plant, and the number of sporulated aphids on each plant are shown. The tab labeled “Figure 2B” includes raw data from the repeat infection after confirming that strain 313 had lost the pRILSR1 plasmid, showing strain, the initial number of aphids on each plant, and the number of sporulated aphids.

SI References

1. Lukasik, P., et al., *Unrelated facultative endosymbionts protect aphids against a fungal pathogen.* Ecol Lett, 2013. **16**(2): p. 214-8.
